# Perovskite Quantum Dots in Solar Cells

**DOI:** 10.1002/advs.202104577

**Published:** 2022-01-14

**Authors:** Lu Liu, Adel Najar, Kai Wang, Minyong Du, Shengzhong (Frank) Liu

**Affiliations:** ^1^ Dalian National Laboratory for Clean Energy iChEM Dalian Institute of Chemical Physics Chinese Academy of Sciences Dalian Liaoning 116023 China; ^2^ University of the Chinese Academy of Sciences Beijing 100039 China; ^3^ Department of Physics College of Science United Arab Emirates University Al Ain 15551 United Arab Emirates; ^4^ Key Laboratory of Applied Surface and Colloid Chemistry Ministry of Education Shaanxi Engineering Lab for Advanced Energy Technology School of Materials Science and Engineering Shaanxi Normal University Xi'an Shaanxi 710119 China

**Keywords:** high efficiency, perovskite quantum dots, solar cells, structure stability

## Abstract

Perovskite quantum dots (PQDs) have captured a host of researchers’ attention due to their unique properties, which have been introduced to lots of optoelectronics areas, such as light‐emitting diodes, lasers, photodetectors, and solar cells. Herein, the authors aim at reviewing the achievements of PQDs applied to solar cells in recent years. The engineering concerning surface ligands, additives, and hybrid composition for PQDSCs is outlined first, followed by analyzing the reasons of undesired performance of PQDSCs. Subsequently, a novel overview that PQDs are utilized to improve the photovoltaic performance of various kinds of solar cells, is provided. Finally, this review is summarized and some challenges and perspectives concerning PQDs are also discussed.

## Graphical abstract

Based on the superior properties of perovskite quantum dots (PQDs) over bulk perovskites, not only the applications of PQDs in perovskite quantum solar cells (PQDSCs), outlining the engineering concerning surface ligands, additives and hybrid composition are reviewed, but also their various roles in other photovoltaic devices, including photo conversion layer, interface layer and additive are presented.

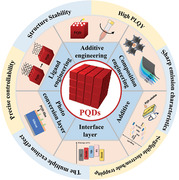

## 1 Introduction

State‐of‐the‐art metal halide perovskites have sparked enormous research attention as promising photovoltaic materials with wide‐range applications in the optoelectronic field. They have been certified to possess excellent carrier migration capability,^[^ ^1^ ^]^ tunable direct bandgap,^[^ ^2^ ^]^ and low exciton binding energy.^[^ ^3^ ^]^ Since Miyasaka et al. advocated perovskite solar cells (PSCs) with a power conversion efficiency (PCE) of 3.8% in 2009,^[^ ^4^ ^]^ the unparalleled “perovskite fever” sweeps the globe and thus far, the certified PCE constantly rising at an unprecedented pace has boosted to 25.5%,^[^ ^5^ ^]^ approximately on par with that of crystal silicon solar cells.^[^ ^6^ ^]^

Perovskite materials have victoriously reaped huge fruits,^[^ ^7^ ^]^ dominantly involving the polycrystalline perovskite films composed of bulky crystal domains. However, both inferior phase stability toward harsh environment (including heat, light, and moisture) and formidable fabrication over a large‐area substrate remain critical challenges hindering the utility‐scale development of PSCs.^[^ ^8^ ^]^ Therefore, on one hand, great efforts have been devoted to improve the perovskite structure stability in parallel with making long‐term operation devices,^[^ ^9^ ^]^ involving ion‐blending strategies to adjust tolerance factor within the range of 0.81–1.11, reinforcing crystallization quality or passivating defects to diminish degradation initiators, preparing stable electrode materials or designing tight encapsulation to insulate external disturbance.^[^ ^10^ ^]^ Be that as it may, these strategies concerning stability are not enough to attain the great goal of commercialization. For instance, the structural stability is still impacted by the volatile and hygroscopic nature.^[^ ^11^ ^]^ On the other hand, quite a few scalable deposition methods including slot‐die coating,^[^ ^12^ ^]^ spray printing,^[^ ^13^ ^]^ vacuum evaporation,^[^ ^14^ ^]^ etc., have been exploited to back up producing PSCs. Nevertheless, significant PCE loss while extending device scale inevitably occurs due to the complex combination of precursor reaction, crystallization plus film formation existing in one fabrication process.

In this case, strikingly, perovskite quantum dots (PQDs), also known as nanocrystals (NCs), are becoming increasingly attractive on account of its various superior properties over bulk perovskites. i) The solvents commonly used for the fabrication of PQD devices are more environmentally friendly non‐polar organic solvents such as octane and hexane, whereas bulk thin‐film perovskites are normally processed from polar aprotic solvents such as *N*,*N*‐dimethylformamide, which is quite toxic.^[^ ^15^ ^]^ ii) PQDs are extremely stable in phase structure as their high surface energy and quantum confinement effect can effectively inhibit the phase transition process,^[^ ^16^ ^]^ so that they can give prolonged life‐span to devices. iii) The absorption spectra and energy levels of PQDs can be easily tuned by size variation, which allows better energy level and absorption matching for PQD‐based optoelectronic devices.^[^ ^17^ ^]^ iv) Since the bandgap of PQDs is formed between Pb lone‐pair s orbital and halogen p orbital, the defects will form only shallow traps enclosed within the conduction or valance band, and will not cause significant monomolecular recombination under excitation.^[^ ^18^ ^]^ So, PQDs enjoy high defect tolerant nature and unique optoelectronic properties like extraordinarily high photoluminescence quantum yield (PLQY), sharp emission characteristics, and negligible electron/hole trapping,^[^ ^19^, ^20^ ^]^ thus potentially conferring photoelectric devices with outstanding performance. Moreover, the multiple exciton effect enable the PQDs of narrow bandgap to exceed the Shockley–Queisser limit, thus achieving superior theoretical efficiency.^[^ ^21^ ^]^ v) Thanks for the decoupling between synthesis of PQDs and film deposition, it is trouble‐free to achieve precise control of PQDs films over the thickness, morphology, and size compared with the perovskite polycrystalline films.^[^ ^19^, ^20^, ^22^ ^]^ For instance, spray‐coating technique effortlessly enables the formation of high‐quality CsPbI_3_ PQDs thin films with a high material utilization ratio.^[^ ^23^, ^24^ ^]^ Thus, PQDs are more compatible with large‐scale flexible devices and tandem devices. Overall, these attractive advantages leave PQDs as preferable materials in the optoelectronic field. In this scenario, the widely application of PQDs in the area of photodetectors,^[^ ^25^ ^]^ lasers,^[^ ^26^ ^]^ and light‐emitting devices^[^ ^27^ ^]^ have been summarized repeatedly. In contrast, a few papers concerning the application of PQDs in the photovoltaic devices have been proposed while they mainly focusing on the perovskite quantum dot solar cells (PQDSCs) and overlooked the other various and paramount functions of PQDs in solar cells.

**Figure 1:**
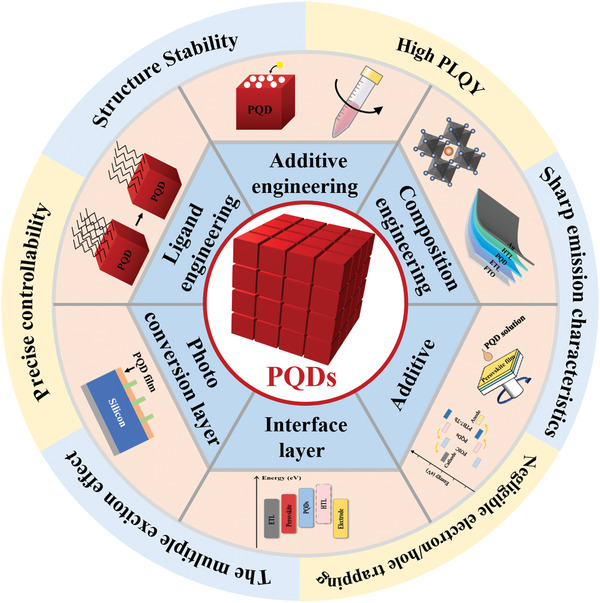
Schematic illustration of properties over bulky crystal domains and applications of PQDs in solar cells.

In this review, we aim to highlight the various functions of PQDs in solar cells with the purpose of directing the development of photovoltaics containing PQDs as shown in **Figure** **1**. In the first section, we aim at presenting the flourishing advancement in PQDSCs, where PQDs are employed as light‐absorbers. In this part, we introduce the ligand exchange strategy of each process according to the preparation of PQDSCs, additive engineering and hybrid PQDSCs. Strikingly, in light of the previous studies, we discuss the reasons of underdeveloped performance of PQDSCs in a penetrating way. Next section covers the applications of PQDs in various solar cells that PQDs are also utilized as photo converser, interfacing materials, and additives to enhance the performance of solar cells, which have been pointed out rarely up to date. Finally, we will end the review with the challenges and prospects of PQDs in future solar cell applications.

## 2 Performance Enhancement of PQDSCs

To date, MAPbX_3_, FAPbX_3_, and CsPbX_3_ QDs with high have been successfully synthesized as well as their hybridization. However, MA‐based PQDs exhibit poor chemical stability due to their low formation energy, limiting its usage as photo absorbers in solar cells. In contrast, although Cs‐ and FA‐based perovskite bulk crystals are metastable at room temperature, their stability is improved upon reducing the crystal size from bulks to quantum dots, rendering their application widespread in photovoltaics. Since the first CsPbI_3_‐based PQDSCs with a PCE of 10.8% reported by Luther et al,^[^ ^28^ ^]^ till now, the highest PCE of PQDSCs has achieved 17.4%, yielded by utilizing Cs_0.25_FA_0.75_PbI_3_ and CsPbI_3_ PQDs as photoabsorbers.^[^ ^29^ ^]^ In the performance enhancing development as summarized in **Table** **1**, a series of optimization strategies were proposed, dominantly involving ligand engineering, additive engineering and hybrid engineering, which will be discussed systemically in this part.

**Table 1:** Summary of the PQDSCs performance (PCE and stability) by different methods

Methods	PQD type	PCE [%]	Stability	Ref.
Ligand engineering	CsPbI_3_ PQDs	10.8%	—	^[^ ^28^ ^]^
	FAPbI_3_ PQDs	8.4%	226 h (continuous illumination) 99% of initial PCE	^[^ ^37^ ^]^
	CsPbI_3_ PQDs	11.9%	—	^[^ ^38^ ^]^
	CsPbI_3_ PQDs	14.6%	—	^[^ ^39^ ^]^
	CsPbI_3_ PQDs	11.2%	1 month (20% RH in air) 80% of initial PCE	^[^ ^24^ ^]^
	CsPbBr_3_ PQDs	4.2%	—	^[^ ^40^ ^]^
	CsPbI_3_ PQDs	12.9%	—	^[^ ^41^ ^]^
	CsPbI_3_ PQDs	14.1%	—	^[^ ^76^ ^]^
	CsPbI_3_ PQDs	13.4%	—	^[^ ^22^ ^]^
	CsPbI_3_ PQDs	14.1%	15 days (ambient condition) > 90% of initial PCE	^[^ ^42^ ^]^
	CsPbI_3_ PQDs	15.2%	—	^[^ ^43^ ^]^
	CsPbI_3_ PQDs	13.7%	10 days (ambient condition) 87% of initial PCE	^[^ ^44^ ^]^
	CsPbI_3_ PQDs	14.3%	7 days (ambient condition) 95% of initial PCE	^[^ ^45^ ^]^
	FAPbI_3_ PQDs	12.7%	—	^[^ ^46^ ^]^
	CsPbI_3_ PQDs	15.1%	—	^[^ ^47^ ^]^
	CsPbI_3_ PQDs	14.9%	—	^[^ ^48^ ^]^
Additive engineering	CsPbI_3_ PQDs	13.1%	7 days (ambient condition) 68% of initial PCE	^[^ ^49^ ^]^
	CsPbI_3_ PQDs	16.1%	10 days (ambient condition) 85% of initial PCE	^[^ ^50^ ^]^
	CsPbI_3_ PQDs	14.8%	50 h (ambient condition) > 95% of initial PCE	^[^ ^51^ ^]^
	CsPbBr_1.5_I_1.5_ PQDs	9.7%	24 days (ambient condition) > 95% of initial PCE	^[^ ^52^ ^]^
	CsPbI_3_ PQDs	12.2%	>90 days (<20% relative humidity, room temperature) 85% of initial PCE	^[^ ^53^ ^]^
	CsPbI_3_ PQDs	11.6%	1 month (N_2_ atmosphere) > 98% of initial PCE	^[^ ^54^ ^]^
	CsPbI_3_ PQDs	15.1% (rigid) 12.3% (flexible)	—	^[^ ^55^ ^]^
	CsPbI_3_ PQDs	16.2%	30 days ≈ 83% of initial PCE	^[^ ^56^ ^]^
	CsPbI_3_ PQDs	12.3%	—	^[^ ^57^ ^]^
Hybrid composition engineering	CsPbBr_1.5_I_1.5_ PQDs	7.9%	35 h (constant exposure to air) 88% of initial PCE	^[^ ^58^ ^]^
	CsPbBr_0.6_I_2.4_ PQDs	12.3%	15 days (ambient condition) 87% of initial PCE	^[^ ^58^ ^]^
	Cs*_X_* FA_1−_ *_X_* PbI_3_ PQDs	16.1%	1000 h (ambient condition) 96% of initial PCE	^[^ ^59^ ^]^
	Cs*_x_* FA_1−_ *_x_* PbI_3_ PQDs	17.4%	—	^[^ ^29^ ^]^
	Cs_1−_ *_x_* FA*_x_* PbI_3_ PQDs	16.6%	600 h (continuous illumination) 94% of initial PCE	^[^ ^60^ ^]^
	CsPbBrI_2_ PQDs	5.3%	—	^[^ ^61^ ^]^
	CsSn_0.6_Pb_0.4_I_3_ PQDs	2.9%	—	^[^ ^62^ ^]^

### 2.1 Ligand Engineering for PQDs

Generally, PQDs can be synthesized by using hot‐injection,^[^ ^30^ ^]^ reprecipitation,^[^ ^31^ ^]^ microfluidic‐assisted synthesis,^[^ ^32^ ^]^ and so on,^[^ ^33^ ^]^ whereas hot‐injection is preferred since it can yield high‐quality PQDs with easily‐tuned size distribution. In the synthesis of PQDs, long‐chain organic moieties such as oleic acid (OA) and oleic amine (OAm) are indispensable because they can modulate reaction dynamics by participating in the metathesis reaction, and absorb on the PQDs surface as capping ligands to maintain stable dispersibility by inhibiting the aggregation coupled with phase transformation.^[^ ^34^, ^35^ ^]^ It is worth noting that the adsorption and desorption of the capping ligands are in a dynamic equilibrium state. Nevertheless, such long insulating organic ligands can form large potential barriers on the PQDs surface, thereby preventing electrical coupling and detrimental to the carrier transport/hopping between adjacent PQDs. Therefore, the synthesis is followed by multi‐post‐treatment process essentially to modulate the capping ligands, i.e., ligand engineering, while ensuring the integrity of PQDs in parallel with precluding aggregation.^[^ ^36^ ^]^ In the following part, we will systematically review the ligand engineering for PQDs including post‐purification process to control the ligand density and ligand‐exchanging processes to regulate ligand species, according to the preparative route of PQD films.

#### 2.1.1 Post Purification for PQDSCs

The post purification process general refers to centrifugal washing procedure following the synthesis, which, in addition to eliminating remnant precursors and screening crystal size, effectively regulate the species and densities of the capping ligands to achieve exceptional photoelectric properties while maintaining excellent phase‐stability for PQDs. This invokes that the solvent polarity used in purification process of PQDs exerts a tremendous influence. Generally, nonpolar solvents can provide a stable colloidal environment for the monodispersed and crystalline PQDs with surface hydrophobic ligands.^[^ ^20^, ^30^, ^34^, ^63^ ^]^ By contrast, polar solvents serving as antisolvents can attack the ligand binding of ionic nature and promote the desorption of surface hydrophobic ligands from PQDs. Then, the underlying perovskite crystal core will aggregate along with phase transformation since perovskites are fully ionic crystals.^[^ ^30^, ^64^ ^]^ Simultaneously, the interaction between PQDs and ligands is also determined by the types of PQDs, which directs the post‐purification solvent selection. As an example, the polar solvent effects on the as‐synthesized CsPbBr_3_ PQDs was ventured.^[^ ^65^ ^]^ Kim et al. mixed as‐synthesized PQDs solution with various polar solvents and established that mildly polar solvents, such as isopropyl alcohol, 1‐butanol, acetone, and acetonitrile enabled CsPbBr_3_ PQDs with proper concentration of ligands to disperse in a stable colloidal form while holding their optical properties. The PQDs are so compatible with various solvents that the post purification process is not the key of PQDSCs research.

**Figure 2:**
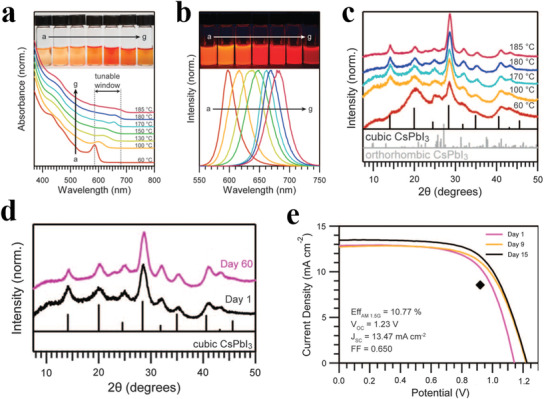
CsPbI_3_ PQD properties and optoelectronic devices. a) Normalized UV–visible absorption spectra and photographs of CsPbI_3_ PQDs. b) Normalized photoluminescence spectra and photographs under UV illumination of the PQDs. c) X‐ray diffraction (XRD) patterns of PQDs. d) Powder XRD patterns of CsPbI_3_ PQDs. e) Current density–voltage (*J–V)* curves of a device measured in air. Reproduced with permission.^[^ ^28^ ^]^ Copyright 2016, American Association for the Advancement of Science.

Actually, CsPbBr_3_ PQDs naturally possess exceptional stability thanks to its appropriate Goldschmidt tolerance factor and strong coupling with ligands,^[^ ^66^ ^]^while the CsPbI_3_ PQDs are more fragile because the softer basic nature of I^−^ as compared with Br^−^ results in vulnerable interactions of I^−^/Pb^2+^, and PQDs/ligands, thus leading to the ease of ligand desorption accompanied by aggregation. Meanwhile, the low Goldschmidt tolerance factor around 0.8 incurs effortless phase transition from black phase (*γ*‐, *β*‐, or *α*‐ phase) to yellow phase (*δ*‐phase) for CsPbI_3_. Therefore, although CsPbI_3_ PQDs have been reported for a few years,^[^ ^30^, ^67^ ^]^ the application in PQDSCs remains challenging due to high susceptibility to purification process. Strinkingly, in 2016, on the basis of hot‐injection methods described by Protesescu et al.,^[^ ^30^ ^]^ Luther et al. not only synthesized CsPbI_3_ PQDs with various sizes by altering injection temperature (**Figure** **2**a–c), but also presented an appropriate post‐purification approach for CsPbI_3_ PQDs by using “magic solution” of methyl acetate (MeOAc) that can remove part of surface ligands without inducing agglomeration.^[^ ^28^ ^]^ In detail, the synthesized PQDs were precipitated by three‐times volume MeOAc, and then re‐dispersed in equivoluminal MeOAc/hexane mixed solvent to screen suitable particle size. Finally, the obtained PQDs dispersed in hexane were refrigerated to remove excess OA, PbI_2_ and by‐products. Noticeably, they unraveled that surface ligands render CsPbI_3_ PQDs stable for months at room even at cryogenic temperature, far below the phase transition temperature of bulk materials. As a result, the devices based on the CsPbI_3_ PQDs showed a promising PCE of 10.8% (Figure 2d,e). Afterward, Shang et al. quantified the effect of post purification on the charge dynamic process at the CsPbI_3_ PQDs/TiO_2_ electron transport layers (ETLs) interface.^[^ ^68^ ^]^ The charge separation time constant in the purified PQDs/TiO_2_ films is 288 ± 1 ps, shorter than that (457 ± 4 ps) in original PQDs/TiO_2_. While the charge recombination time constants raised from 346 ± 18 ns to 1180 ± 60 ns upon post purification. This result demonstrated partially removing native ligands on PQDs can increase charge separation efficiency and simultaneously reduce charge recombination loss.

**Figure 3:**
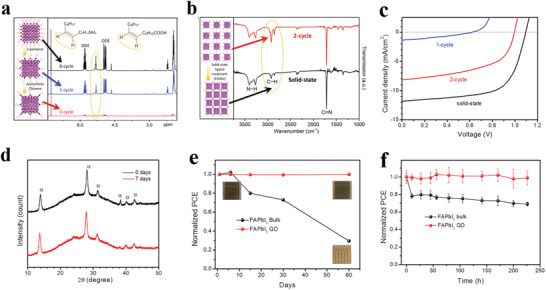
a) Nuclear magnetic resonance spectra of FAPbI_3_ PQDs with increasing number of surface treatment cycles. b) Fourier transform infrared (FTIR) spectra of FAPbI_3_ PQDs. c) *J–V* curves of the devices. d) XRD patterns of FAPbI_3_ PQD films. e) Evolution of PCE of devices based on bulk FAPbI_3_ and FAPbI_3_ PQDs. f) PCE change of the encapsulated device under continuous illumination. Reproduced with permission.^[^ ^37^ ^]^ Copyright 2018, Elsevier.

In comparison with CsPbX_3_ PQDs, FAPbI_3_ PQDs are more desirable because of the following advantages. i) FAPbI_3_ possesses better charge transport property due to the faster formation of large polaron in the organic‐inorganic hybrid perovskites than in its inorganic counterpart.^[^ ^69^ ^]^ ii) FAPbI_3_ enjoys the narrowest bandgap among the lead halide perovskites, then FAPbI_3_ PQDs still possess a desirable absorption edge at around 800 nm in spite of quantum confinement effect.^[^ ^70^ ^]^ iii) Compared with CsPbI_3_ (≈0.85), the Goldschmidt tolerance factor of FAPbI_3_ (≈1.03) is closer to 1, which may cause FAPbI_3_ PQDs to possess better phase stability (stable for at least several months).^[^ ^71^ ^]^ However, the chemical binding between FA^+^ and [PbI_6_] octahedra in FAPbI_3_ is weaker than the analogue in CsPbX_3_,^[^ ^72^ ^]^ so dealing with the capping ligands for FAPbI_3_ PQDs is more formidable. To address this issue, Yang et al. customized a post purification process consisting of three procedures for FAPbI_3_ PQDs.^[^ ^37^ ^]^ First, a protic solvent of 2‐pentanol with relative polarity index (RPI) of 0.488 was introduced into the PQDs solutions to remove large numbers of ligands and also, the protic character can stabilize FA^+^, maintaining the ionic lattice structure of FAPbI_3_. Then, a mixed solvent that acetonitrile and toluene was at the volume ratio of 4:3, whose RPI is equal to 0.243, was utilized to further lower ligand density for PQDs. Finally, ethyl acetate (EtOAc) with a lower RPI of 0.228, was applied to rinse the FAPbI_3_ PQD films. In so doing, the concentration of surface ligands on PQDs was reduced adequately (**Figure** **3**a,b). Thereby, the inter‐dot electrical coupling is significantly enhanced while maintaining the integrity of FAPbI_3_ PQDs. As such, the PQDSCs not only presented a positive PCE of 8.4%, but also exhibited preferable ambient and operational stability over their bulk counterpart (Figure 3c–f).

#### 2.1.2 Liquid‐State Ligand Exchanging of PQDSCs

Apart from the efforts on lowering ligand density by altering post purification process, exchanging raw ligands (e.g., OA and OAm) with shorter species is another effective approach in order to compensate the undeveloped charge transfer capability triggered by surface ligands.^[^ ^73^ ^]^ Since the preparation of PQDs comprise two processes, namely, synthesis and post purification, the specific ligands can be introduced in a certain process. As for in situ introducing ligands in the synthesis process, basing on the referenced capping ligands of OA and OAm, Chen et al. partially substituted them by using shorter ligands of octanoic acid (OctAc) and octylamine (OctAm).^[^ ^38^ ^]^ Benefiting from their higher polarity, the resulting stronger interaction between CsPbI_3_ and OctAc/OctAm produces larger PLQY, higher charge transport rate and better stability compared to the counterpart using OA/OAm. As a result, PQDSCs exhibit an enhanced PCE from 7.8% to 11.9% upon partial substitution of ligands. Similarly but more strikingly, Ma et al. incorporated L‐phenylalanine (L‐PHE) with a bifunctional ligand into the precursor solution during synthesis. The strong coordination with both the cation and ions on PQDs surface can significantly reduce surface states and increased vacancy formation energy, thereby raising the PLQYs, accelerating chare transfer and improving stability. Eventually, L‐PHE passivated CsPbI_3_ PQDSCs realized an optimal PCE of 14.6%.^[^ ^39^ ^]^

As mentioned above, exchanging ligands can also be executed in post‐purification process. Tian and co‐workers successively introduced short‐chain ligands of phenyltrimethylammonium bromide (PTABr) and 2‐aminoethanethiol (AET) to partially substitute OA/OAm.^[^ ^24^, ^74^ ^]^ Taking PTABr as an example, the amine ions from PTABr can adhere to halide ions of PQDs whereas the Br^−^ can diffuse into the I^−^ vacancy of PQDs, both passivating defects and forming a hydrophobic surface. Additionally, armed with shortened length between adjacent PQDs, this process can also enhance the carrier charge mobility within the PQDs films. Thus, PQDSCs using PTABr displayed a competitive PCE of 11.2% with an improved long‐term stability.

#### 2.1.3 Solid‐State Ligand Exchanging for PQDSCs

**Figure 4:**
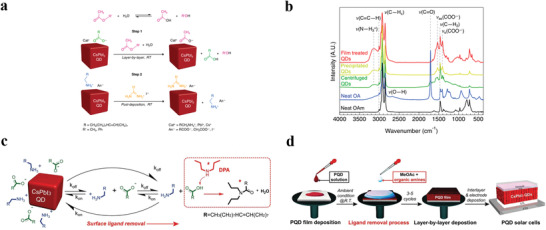
a) Schematic of ligand exchange in CsPbI_3_ thin films. b) FTIR spectra of neat OAm (black), OA (blue), and CsPbI_3_ PQD films (green, amber, and red). Reproduced with permission.^[^ ^75^ ^]^ Copyright 2018, American Chemical Society. c) Schematic of the CsPbI_3_ PQD dynamic surface stabilization and the ligand removal process. d) Diagram of the fabrication of devices based on CsPbI_3_ PQDs. Reproduced with permission.^[^ ^48^ ^]^ Copyright 2020, Wiley‐VCH.

Following the synthesis and post‐treatment processes, the as‐obtained PQDs are deposited in a layer‐by‐layer fashion to form active layers for PQDSCs. For instance, PQD solution was usually spin‐coated on substrates for 3–5 times to achieve an PQD film with sufficient thickness of ≈200–500 nm. In this process, since an additional PQD layer partially eliminates the underlying layers, thus solid‐state ligand exchanging targeting adjacent PQD layers is indispensable to prevent the re‐dissolving issue in parallel to further enhance the coupling between PQDs. Analogously, esters such as MeOAc and EtOAc are coated on the surface of PQDs, acting as antisolvents to remove OA ligand through anion change in this procedure. Strikingly, Wheeler et al. revealed the MeOAc‐assisted ligand exchanging process on PQD surface at a molecular‐level (**Figure** **4**a) and pointed out the amount of adventitious water was paramount for preparing high‐efficiency PQDs.^[^ ^75^ ^]^ In detail, MeOAc hydrolyzes with adventitious water and the produced acetic acid and methanol float around in the mixture. Then, acetic acid molecules protonate oleate ligands and replace native ligands, yielding PQD surface‐bound acetate and free OA (Figure 4b).^[^ ^75^ ^]^ In this manner, the distance between PQDs was shortened, thus cranking up the electron coupling and facilitating carrier transportation.

And, Kim's group devised a suitably optimized solvent mixture of EtOAc/BuOAc system to enable efficient ligand exchange and simultaneously to suppress stripping‐out phenomena of the CsPbBr_3_ PQD bottom layer resulting from high miscibility with hydrophobic substances.^[^ ^40^ ^]^ The solvent miscibility‐induced solid‐state ligand exchanging enabled the fabrication of thick and pinhole‐free CsPbBr_3_ PQD films and improved the PCE of CsPbBr_3_ PQDSCs up to 4.23% with a high open‐circuit voltage (*V*_OC_) of 1.59 V. However, since it has to make a trade‐off between removing long‐chain organic ligands for high charge transport and keeping them for the stabilization of the optically active crystal phase, related to the usage times and volume of MeOAc, the reproducibility for PQDSCs using antisolvent, such as MeOAc, treatment is poor. To explore the treatment of MeOAc specifically, Han et al. controlled the treatment degree of MeOAc by the cycle times. They demonstrated that the device undergoing three‐cycle MeOAc treatment can achieve the highest PCE of 12.85% staying phase stability.^[^ ^41^ ^]^

However, the pure solvent treatment brings about some shortcomings. First, the ligand exchanging rate and degree depend on the amount of adventitious water as well as the extent of ester hydrolyzation. Second, as another dominative capping ligand, OAm is overlooked in the pure esters. Last, the ligand exchanging generate amounts of charged surface vacancies and dangling bonds that further capture photons as defect states and result in defect‐assisted non‐radiative recombination in PQDs.^[^ ^76^ ^]^ In addition, the hydrolysis of MeOAc would cause undesired metal hydroxide formation, while the Pb‐OH formation yields sub‐bandgap trap states resulting in low charge transport.^[^ ^77^ ^]^ Also, acidic condition followed by the hydrolysis make the perovskites suffer from lattice distortion.^[^ ^78^ ^]^ Therefore, appropriate reagents are required in solvents to provide compact surface species upon replacing the native ligands.

Typically, a series of ionic salts including acetates,^[^ ^28^, ^78^, ^79^ ^]^ nitrates,^[^ ^28^, ^79^ ^]^ and halides^[^ ^80^ ^]^ were utilized and the corresponding cations are generally alkali metal ions, Pb^2+^, FA^+^, and MA^+^. The beneficial functions are various depending on the ionic combination. In detail, Ac^−^ can accelerate ligand exchanging, and enhance the electron coupling since it is an appropriate surface ligand as mention above.^[^ ^78^ ^]^ Moreover, compared with acetic acid generated by hydrolysis of MeOAc, OAc^−^ devoid of protons can reduce the solution polarity and minimize the destroy on crystal structure. In terms of cations, Pb^2+^‐ and Cs^+^‐based salts can also fill the vacancy triggered by ligand exchanging.^[^ ^28^, ^79^ ^]^ Additionally, FA^+^ is preferred since it can exchange with native OAm ligands. For example, Sanehira et al. presented an AX post treatment (where A = FA^+^, MA^+^, or Cs^+^ and X = I^−^ or Br^−^) whereby each PQD layer was successively immersed in a saturated AX salt solution and neat EtOAc for ≈10 s. In this process, the acetic acid molecules produced by the hydrolyzation of EtOAc replace oleate ligands and A^+^ is responsible for replacing OAm. Thus, all the AX salt treatments markedly increased the performance of the CsPbI_3_ PQD films by greatly improving the electronic coupling between PQDs and enhances carrier mobility in films.^[^ ^22^ ^]^ Among the AX salts, FAI‐modified devices displayed the highest PCE of 13.4% due to the increment of *J*_SC_ and noticeable reduction in hysteresis.

However, the FAI/MeOAc solution usually results in poor device stability due to the hygroscopic FA^+^ and excess FA^+^ hybridized on the surface and inside of the PQDs will lead to undesired decrease in energy gap and reduce *V*_OC_. Therefore, aromatic‐ring‐ based phenylethanamine iodide (PEAI)^[^ ^42^ ^]^ and guanidinium thiocyanate (GASCN)^[^ ^43^ ^]^ were successively incorporated to treat CsPbI_3_ PQDs for removing OAm ligands. Their much shorter carbon chain than OAm and hydrophobic feature enable both strong electronic PQD coupling and high moisture stability. As a result, PQDSCs prepared with PEAI and GASCN treatments yielded high PCEs of 14.1% and 15.21%, respectively.

Besides, small organic molecules such as zwitterionic amino acids,^[^ ^44^ ^]^ pyridine derivatives^[^ ^45^ ^]^ and non‐fullerene electron accepters^[^ ^46^, ^47^ ^]^ can also tune the surface chemistry of PQDs since their functional groups can form bonds with the CsPbI_3_ PQD surface, thereby effectively passivating defects. In addition to the passivation effect, special influence is also introduced determined by the molecule structures. The amino acids possessing functional groups of carboxyl (RCOO^−^) and ammonium (RNH_3_^+^) are also conductive to remove the long chain ligands through ligand exchange.^[^ ^44^ ^]^ Besides, organic electron acceptors involving conjugated small molecule, 2,2′‐[[6,6,12,12‐tetrakis(4‐hexylphenyl)‐6,12‐dihydrodithieno[2,3‐d:2′,3′‐d′]‐s‐indaceno[1,2‐b:5,6‐b′]dithiophene‐2,8‐diyl]bis[methylidyne(3‐oxo‐1H‐indene‐2,1(3H)‐diylidene)]]bis[propanedinitrile] (ITIC),^[^ ^46^ ^]^ and non‐fullerene Y6‐F^[^ ^47^ ^]^ can develop a hybrid heterointerface, providing an additional driving force for effective charge separation. For example, a type‐II energy level alignment was formed by the CsPbI_3_ PQD/Y6 heterointerfaces, thus enabling efficient charge transfer/extraction as well as boosting the PCE to 15.05%.

In special, Wang et al.^[^ ^48^ ^]^ utilized di‐*n*‐propylamine (DPA)/MeOAc solution to accelerate the desorption of OA, OAm due to a distinct mechanism as follow: native ligand of OA^−^ and OAm^+^ fall off the surface and react with each other to generate OA and OAm reversibly and dynamically. Then DPA can induce acylation reaction with OA without any heating treatment, which propels the ligand desorption (Figure 4c). Therefore, this solid‐state treatment efficiently removed both long insulating surface ligands of OA and OAm (Figure 4d). Resultantly, the performance of CsPbI_3_ PQDSC improved greatly, owing to the enhancement of electrical coupling and reduced charge recombination, approaching a PCE of 14.9%. The treatment of DPA provides a gentle and efficient approach for the control of the surfaces ligand density of PQDs, and emphasizes the meaning of creating novel ligand‐management strategies.

### 2.2 Additive Engineering of PQDSCs

**Figure 5:**
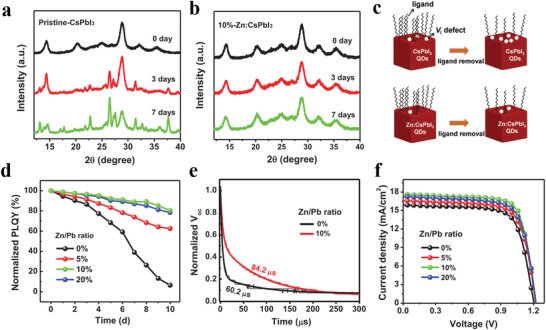
XRD patterns of a) pristine CsPbI_3_ and b) 10%‐Zn:CsPbI_3_ PQDs with storage for different days. c) Schematic illustration of I defect state (V_I_) control by ZnI_2_. d) The variation of PLQY values as a function of aged days of the corresponding films. e) Transient photovoltage curves of pristine CsPbI_3_ and 10%‐Zn:CsPbI_3_ PQDSCs. f) *J*–*V* curves of the fabricated devices. Reproduced with permission.^[^ ^50^ ^]^ Copyright 2020, Wiley‐VCH.

Drawing on the optimizing experience from perovskite polycrystalline films, introducing additives (including extrinsic ion doping) ought to be a promising approach to control the photoelectric property for PQDs. As for the ion doping, a lot of work concerning light‐emitting applications, has verified that dopants of lanthanide ions, Zn^2+^, Sr^2+^, tends to endow PQDs with enhanced optical property, especially increased PLQY.^[^ ^81^ ^]^ For photovoltaic applications, Ma et al. doped 20% ytterbium (Yb) into CsPbI_3_ PQDs and demonstrated that it effectively reduces the trap states by filling lattice vacancies and by increasing crystallinity, thus improving PLQY, charge transport ability and thermal stability of PQDs. PQDSCs based on Yb:CsPbI_3_ PQDs achieved a high PCE of 13.1%.^[^ ^49^ ^]^ Besides, zinc halides (ZnI_2_ and ZnCl_2_) were also employed as dopants to synthesize Zn:CsPbI_3_ PQDs.^[^ ^50^, ^51^ ^]^ Zn^2+^ doping increases the Goldschmid factor of perovskite lattice as well as the formation energy, thereby favoring excellent stability (**Figure** **5**a,b), while the additional halide ions help to mitigate the iodine vacancy to achieve higher PLQY (Figure 5c,d). As a consequence, the charge recombination loss in PQDSCs is lessened (Figure 5e) and strikingly, Zn:CsPbI_3_ PQDSCs provided a high PCE exceeding 16%, which is one of the highest efficiency of pure PQDSCs up to now (Figure 5f).^[^ ^50^ ^]^ Furthermore, since Ag^+^ possesses an ionic radius of (115 pm), closer to Pb^2+^ (119 pm), Ghosh et al. incorporated Ag^+^ into CsPbBr_1.5_I_1.5_ PQDs^[^ ^52^ ^]^ and deemed it will not perturb the crystal structure significantly.^[^ ^82^ ^]^ With the PQDs having 3.5 atom % Ag^+^, a significant ≈20% enhancement in PCE and ambient stability are observed for the reason that the reduction of surface and intrinsic defects, and the decrement in nonradiative recombination leads to an increased carrier lifetime in parallel with an enhanced charge transfer process.

Noticeably, in contrast to bulk perovskites, the extrinsic ion doping for PQDs is highly selective. Liu et al. adopted GeI_2_ in conjunction with trioctylphosphine as solvent to substantially substitute the dosage of PbI_2_. But noticeably, Ge^2+^ level in CsPbI_3_ PQDs is negligible as well as its effect on the photoelectrical property.^[^ ^53^ ^]^ Videlicet, Ge^2+^ is incapable of substituting Pb^2+^ in PQDs. This result also implies that the excess iodide ions are more valuable whilst Pb‐rich conditions are secondary for getting high‐quality PQDs.

In addition to extrinsic ions, a few additives were also incorporated in the PQD solution directly to improve the performance of films. Since the electrical resistance in PQD films is pretty large and impedes the charge transport efficiency, our group applied micrometer‐sized graphene (μGR) sheets to link CsPbI_3_ PQDs through hydrogen bonds because μGR possesses plenty of functional groups, such as hydroxyl, carbonyl, carboxyl and so on.^[^ ^54^ ^]^ Thanks to the super high electron mobility of μGR sheets, photogenerated electrons extracted from PQDs to the μGR can pass along swiftly. At the same time, the μGR acts as a spacer preventing PQDs from aggregating, blocking the phase transition. Moreover, the hydrophobic character of μGR can also hold back water molecules so that the moisture stability of μGR/CsPbI_3_ films is improved with water contact. Naturally, the device based on μGR/CsPbI_3_ PQDs performs enhanced PCE of 11.6% and improved stability under ambient environment and thermal stress. Besides, Hu et al. took phenyl‐C_61_‐butyric acid methyl ester (PCBM) into the CsPbI_3_ PQD solutions.^[^ ^55^ ^]^ PCBM bonds with the undercoordinated Pb^2+^ ions on the PQD surfaces through functional carboxyl groups and forms an exciton cascade between CsPbI_3_ PQD layer and ETLs. Consequently, both the PQD heterointerfaces and the PQD/ETL interfaces achieve effective charge transfer and efficient exciton dissociation. As such, rigid and flexible PQDSCs present champion PCEs of 15.1% and 12.3%, respectively. To diminish surface defects, Zhang and his group introduced a “surface matrix curing” (SMC) strategy where they added nucleophile trioctylphosphine (TOP) to a hexane solution containing tert‐butyl iodide (TBI), which was used to dissolve the once‐purified PQDs.^[^ ^56^ ^]^ The sufficient iodide ions produced by the unimolecular nucleophilic substitution reaction between TBI and TOP fill in the iodide vacancies of the PQD surface matrix, and then the nonradiative recombination was substantially reduced. Consequently, the PQDSCs yielded a PCE of 16.2%, which is the record value among inorganic devices based on CsPbI_3_ PQDs. Benefiting from filling ionic vacancies, which could act as attack sites for moisture and oxygen to accelerate degradation,^[^ ^83^ ^]^ the stability of PQDSCs was also extensively enhanced. Recently, Yuan's team introduced an organic dopant 2,2′‐(perfluoronaphthalene‐2,6‐diylidene) dimalononitrile into CsPbI_3_ PQDs to realize the carrier‐type transformation of PQD arrays from n‐type to p‐type.^[^ ^57^ ^]^ Then the P/N homojunction PQDSC was successfully assembled using different carrier‐type PQDs, delivering an enhanced PCE of 15.3%. Most importantly, it showed a record high efficiency of 12.3% for a 1.2 µm thick PQD active‐layer, demonstrating great potential for the future printing manufacturing of PQDSCs.

### 2.3 Hybrid Composition Engineering of PQDSCs

Motivated by the composition engineering of bulk perovskite polycrystals, finely tuning the chemical composition of PQDs and forming hybrid species also give access to both structural and optical quality enhancements in comparison with pure counterparts. For instance, theoretical studies illustrate that half substitution of I^−^ by Br^−^ ions raises the ion diffusion barrier and stabilizes the structure of perovskite.^[^ ^84^ ^]^ Similar to bulk perovskites, the hybrid PQDs can be achieved by mixing halide salt precursors in the reactors. Comparatively early, Protesescu et al.^[^ ^34^ ^]^ synthesized CsPbX_3_ (X = Cl, Br, and I) PQDs through modulating lead halide composition in precursors and obtain emission spectra covering entire visible spectral region of 410−700 nm. Furthermore, Ghosh et al. and Liu et al. synthesized CsPbBr*_x_* I_3−_ *_x_* (*x* = 3–1) PQDs by adding PbBr_2_ and PbI_2_ at the certain ratio.^[^ ^58^ ^]^ The CsPbBr_1.5_I_1.5_ had the advantage of improved stability under ambient condition, and highest average lifetime compared with other CsPbBr*_x_* I_3−_ *_x_* PQDs.

However, the formation of certain PQDs compositions is not only governed by the thermodynamics of the mixed‐ion PQDs, but also by the PQD surfaces (i.e., surface energy and ligand binding) and chemical equilibria with the precursors in the solution. These factors greatly expand relevant parametric space beyond the mixing ratios of precursors. As an example, Kovalenko et al. mixed FA‐oleate and Cs‐oleate in precursors to obtain FA*_x_* Cs_1−_ *_x_* PbI_3_ PQDs, yet only FA_0.1_Cs_0.9_PbI_3_ can be synthesized regardless of FA/Cs ratio in precursors.^[^ ^85^ ^]^ Thus, for the precursor‐mixing approach, it is formidable to control the final product due to differences in precursor reactivity, unless utilizing specially equipment, such as microfluidic platform.^[^ ^86^ ^]^

To address this issue, ionic exchanging was proposed to gives access to prepare A‐site hybrid PQDs. While simply mixing two PQDs of different compositions or mixing PQDs with ion precursors in medium solutions, an efficient vacancy‐assisted ion diffusion/exchange occurs with the preservation of shape and crystal structure of the parent PQDs. This process builds on platform of PQDs with surface vacancies, dynamic surface ligands, a high surface‐to volume ratio, and the rigid cationic sublattice. Park et al simply stacked CsPbI_3_ PQD film on the FAPbI_3_ PQD thin film and uncovered that the solid‐state ion‐exchange transformation occurs slowly. The formed Cs*_X_* FA_1−_ *_X_* PbI_3_ phase with a graded band structure and entropic stabilization improved both device performance and stability.^[^ ^59^ ^]^ Luther's group presented liquid‐state cation‐exchange approaches for tunable hybridization of CsPbI_3_ and FAPbI_3_ PQDs that enables the formation of compositions spanning the complete range of Cs_1−_ *_x_* FA*_x_* PbI_3_, showing bright and finely tunable emission in 650–800 nm range.^[^ ^18^ ^]^ The activation energy for the miscibility between Cs^+^ and FA^+^ is as high as 0.65 eV, thus the A‐site alloy usually occurs at elevated temperature rather than room temperature. They demonstrated replacing Cs^+^ with FA^+^ can increase the carrier lifetime and charge‐carrier mobility in PQDs,^[^ ^87^ ^]^ because the fast rotation of FA^+^ results in enhanced orbital overlap and easier polaron formation.^[^ ^69^, ^88^ ^]^ The devices based on the hybrid PQDs exhibit a lower *V*_OC_ loss than the thin‐film perovskite devices of similar bandgap due to the higher defect‐tolerant capability of the former. Since a higher FA^+^ content leads to deeper band positions relative to vacuum compared to pure CsPbI_3_ PQDs, they achieved to fabricate PQDSCs with abrupt composition/energy band changes throughout the PQDs film by using the layer‐by‐layer strategy, where the FA*_x_* Cs_1−_ *_x_* PbI_3_ and CsPbI_3_ PQDs are employed as the underlayer and upper layer respectively. In this scenario, the created internal heterojunction facilitates charge separation and improves photocarrier harvesting. The PQDSCs based on the bilayer absorber achieved a high PCE of 17.4% with a stabilized power output of 15.5%.^[^ ^29^ ^]^

**Figure 6:**
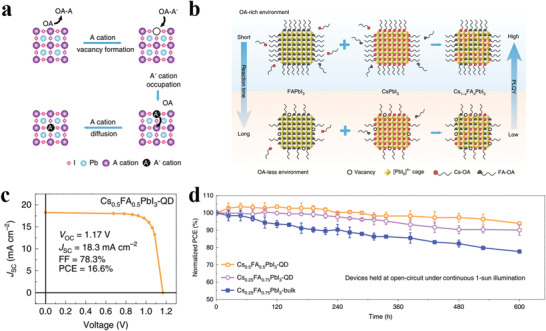
a) Proposed A‐site cation‐exchange reaction mechanism. b) Schematic illustration of cation exchange in different environments. c) Certificated *J–V* curve. d) Stability of unencapsulated solar cells fabricated with Cs_0.25_FA_0.75_PbI_3_‐bulk film, Cs_0.25_FA_0.75_PbI_3_‐PQD film, and Cs_0.5_FA_0.5_PbI_3_‐PQD film. Reproduced with permission.^[^ ^60^ ^]^ Copyright 2020, Springer Nature.

Apart from temperature‐driven cation exchange between CsPbI_3_ and FAPbI_3_ PQDs, Wang et al. discovered increasing the surface capping ligand density can also accelerate the cation‐exchange dynamics while maintaining high radiative efficiency by suppressing surface defects (**Figure** **6**a,b).^[^ ^60^ ^]^ Microscopically, the FA^+^ and Cs^+^ redistributed among the corner‐sharing PbI_6_ octahedrons, growing into small FAPbI_3_ and CsPbI_3_ units and macroscopically forming single cubic‐shaped Cs_1−_ *_x_* FA*_x_* PbI_3_ PQDs. The mixed Cs_1−_ *_x_* FA*_x_* PbI_3_ (*x* = 0–1) PQDs exhibit superior stability and charge transport properties to pure CsPbI_3_ and FAPbI_3_ PQDs. Among them, Cs_0.5_FA_0.5_PbI_3_ PQDSCs presented the highest PCE of 16.6% with negligible hysteresis and noticeably, the devices exhibit outstanding photostability whereby retaining 94% of the original PCE after continuous illumination for 600 h (Figure 6c,d). Videlicet, the notorious photoinduced phase segregation in the case of high Cs ratio is suppressed in PQDs, compared with bulk phase film, which can be attributed to the suppressed ion migration and the confinement effect in PQD films.^[^ ^89^ ^]^

In addition to the A‐site cation exchanging, B‐ and X‐site exchanging is also available. In comparison with the hour‐scale A‐site exchange that must be accelerated by elevating temperature or increasing population of capping ligands, X‐site exchange generally occurs rapidly at a minute scale even at room temperature. And noticeably, A‐site mixed PQDs can only be obtained by alloying various PQDs rather than mixing PQDs with A‐contained precursors because the latter will introduce additional reaction in parallel with generating by‐product,^[^ ^53^ ^]^ while X‐site exchanging is flexible as it can be achieved by mixing CsPbX_3_ PQDs with halide precursors (ammonium halides and lead halides) or CsPbX_3_ PQDs. Analogously, the obtained X‐site hybrid PQDs with excellent structure integrality and stability exhibited comparable optical quality to that of directly synthesized PQDs.^[^ ^20^, ^34^ ^]^ With this in mind, Christodoulou et al. performed anion exchange reactions in concentrated CsPbBr_3_ PQDs solutions with PbI_2_ solution, thus inducing the formation of CsPbI_2_Br PQDs obtaining a high PLQY of 65% in films and a PL red shift up to 676 nm. Then PQDSCs, which were fabricated in the wavelength range 350−660 nm, were operated in air and displayed a PCE of 5.3% with a *V*_OC_ up to 1.31 V.^[^ ^61^ ^]^ As for the B‐site exchanging, Shen et al. alloyed CsSnI_3_ PQDs with CsPbI_3_ PQDs to synthesize CsSn_1−_ *_x_* Pb*_x_* I_3_ PQDs for the purpose of extending bandgap and reducing Pb usage, notably, which is structure‐stable for long time in purified colloidal solution and remains structure intactness even directly exposed to ambient air, showing far superior stability to its parent of CsSnI_3_ and CsPbI_3_ PQDs.^[^ ^62^ ^]^ Furthermore, the fast electron transfer rate from PQDs to TiO_2_ imply the potentially high PCE of solid‐state heterojunction devices if the residual ligands on PQD surface can be controlled appropriately.

### 2.4 Some Analysis in PQDSCs

PQDSCs have presented distinguishing superiorities over the composition regulation, film deposition, and device stability. However, the PCE of PQDSCs is still far behind that of the bulk PSCs. Then we will analyze the energy loss in PQDSCs from two perspectives, that is, deficit photovoltaic parameters and carrier dynamic process.

#### 2.4.1 Analysis from the Deficit Photovoltaic Parameters

PQDSCs enjoy comparable *V*_OC_^[^ ^90^ ^]^ and FF while the inferior *J*_SC_. According to the definition of external quantum yield, *J*_SC_ is related to the product of light harvesting efficiency (i.e., absorptance, *η*_abs_), electron injection efficiency (*η*_inj_), and charge collection efficiency (*η*_coll_). The *η*_coll_ is considered to be acceptable as it is determined by the CTLs, regardless of PQDs. Meanwhile, the *η*_inj_ for PQDSCs is also appropriate which will be discussed in the following part. Nevertheless, the *η*_abs_ in PQDSCs is insufficient because the thickness of PQDs films is inadequate with the purpose of enabling fluent charge transport process and ensuring high FF, which is regarded the dominantly barrier degrading *J*_SC_.

The theoretical highest *V*_OC_ is equal to internal quasi Fermi level splitting (QFLS), which is determined via the bandgap and the PLQY. Although the PLQY of PQDs, about 90%,^[^ ^91^ ^]^ is much higher than bulk perovskites, about 20%,^[^ ^92^ ^]^ and the quantum confinement effect extends the bandgap, the *V_OC_* of PQDSCs is not higher than that of bulk PSCs. In view of the *V*_OC_ loss analysis of bulk perovskite and chalcogenide colloidal quantum dot solar cells via detailed balance theory,^[^ ^93^, ^94^ ^]^ we conclude the *V*_OC_ loss of PQDSCs originating from the following factors: i) various defects form in the processes of synthesis and ligand exchange of PQDs. ii) Within the PQDs, ligands and mobile ions give rise to internal voltage drops that generate non‐radiative energy loss channels. iii) Band misalignment results in the voltage drops. iv) Nonradiative recombination at the perovskite/transport layer interfaces and within the transport layers.^[^ ^95^ ^]^ Therefore, fabricating superior PQD film quality with less defects, and optimizing the interfacial contact and energy band alignment between PQD layers and carrier transport layers (CTLs) are of great significance for achieving low *V*_OC_ loss in PQDSCs.^[^ ^93^ ^]^ For example, Yuan et al. achieved CsPbI_3_ PQDs of three bandgap values (1.78, 1.79, and 1.80 eV) by controlling the size of the PQDs and then constructed gradient‐band homojunction solar cells, thus inducing the formation of additional driving force for the carries, which facilitates the charge extraction and increases the carrier diffusion length in the PQD film.^[^ ^96^ ^]^ Consequently, the device yielded a high PCE of 13.2% with *V*_OC_ as high as 1.25 V.

#### 2.4.2 Analysis from the Carrier Dynamic Process

The carrier dynamic process of photovoltaic conversion comprises the following three steps, i) photogenerated exciton breaks up into free carriers that then diffuse within PQDs films due to entropy effect; ii) the free carriers transfer from PQDs to CTLs, named as charge injection or charge separation process; iii) the injected carriers in CTLs transport to electrodes. Then we will discuss point by point to further analyze the energy loss in PQDSCs.

As for the step i, the exciton binding energies of PQDs was estimated to be ten times higher than that of the bulk counterparts, thus impeding generation of free carriers.^[^ ^85^, ^97^ ^]^ In the context of conventional quantum dot solar cells, designing core‐shell structure, for example, a CdTe/CdSe type‐II core/shell structure, is an effective strategy to facilitate charge separation process, which is not compatible with PQDSCs till now.^[^ ^98^ ^]^ Therefore, strategically suppressing the exciton recombination in the PQD films still remains a challenge waiting to be addressed. The carrier transport capability of PQD films is determined by the ligand management on the PQD surface due to the inter‐dots charge hopping mechanism.^[^ ^99^ ^]^ Although MeOAc as a successful antisolvent capably removes large amount of OA from PQDs, amines become the dominative capping ligands whilst this abundant moiety is generally ignored.^[^ ^35^ ^]^ Thus the ligand exchanging engineering toward OAm should be further advanced.

As for step ii, the charge transfer dynamics between PQD and CTLs are attribute to the difference in energy level, the interface coupling degree and density of accepting states, according to Marcus theory. In this step, the hole injection dynamics is so fast that its effect on whole charge transport process is insignificant, especially for organic hole‐transporting materials (HTMs).^[^ ^100^ ^]^ This phenomenon may be specially related to the strong coupling between PQDs and HTM generally possessing various functional groups. Furthermore, the hole injection dynamics can be enhanced by forming polymer‐PQD hybrid bulk heterojunction.^[^ ^101^ ^]^ As for the PQDs/electron transport layers interface, armed with Marcus theory, Liu et al. demonstrated that the electron injection efficiency from CsPbI_3_ PQDs into mesoporous TiO_2_ layers is as high as 99% with an injection rate up to 2.1 × 10^10^ s^−1^.^[^ ^102^ ^]^ Chen et al. further demonstrated that the electron injection rate constants from PQDs to mesoporous TiO_2_ and compact TiO_2_ are comparable.^[^ ^103^ ^]^ And, Ding et al. presented that the photoexcited carrier injection efficiencies at the FAPbI_3_ PQD/TiO_2_ and FAPbI_3_ PQD/NiO*_X_* heterojunctions are found to be as high as over 99% with the rate of 2.01–2.29 × 10^9^ s^−1^ and 1.55–1.96 × 10^9^ s^−1^, respectively.^[^ ^104^ ^]^ Thus, the influence of charge separation progress on the performance of PQDSCs is minor, at least at this stage. Apart from the high density of conduction‐band states of TiO_2_,^[^ ^105^ ^]^ the fast electron‐transfer rate from PQDs to TiO_2_ is attributed to their large offset in energy levels, notably, which in turn triggers large voltage loss in devices.

In terms of the step iii, there are an ocean of alternative materials that have been developed for bulk PSCs, whereas only a few charge transport materials are developed for PQDSCs. For example, TiO_2_ dominates the electron transport materials for PQDSCs, although its significantly poor electron mobility of 0.1–4 cm^2^ V^−1^ s^−1^ limits the device efficiency of and susceptibility to ultraviolet irradiance accelerate the degradation of perovskites.^[^ ^106^ ^]^ Thus, the compatibility of non‐TiO_2_ materials with PQDs is in urgent need of reveal. As a special example, Kim et al. developed chloride‐passivated SnO_2_ QDs ETLs in CsPbI_3_ PQDSCs. The Cl@SnO_2_‐based devices showed improved *V*_OC_ and *J*_SC_, resulting in enhanced PCE up to 14.5% compared to that of TiO_2_‐based control devices with the PCE of 13.8%. And, building on the low hydrophilicity and photocatalytic activity, the device stability was improved.^[^ ^107^ ^]^ As for HTMs, poly(triarylamine) (PTAA) and 2,2,7,7‐tetrakis‐(*N*,*N*‐di‐p‐methoxyphenylamine)‐9,9‐bifluorene (spiro‐OMeTAD) are commonly utilized for PQDSCs. However, they require complex doping and oxidation processes, notoriously triggering device instability. To avoid these drawbacks, dopant‐free poly[4,8‐bis[(2‐ethylhexyl)oxy]benzo[1,2‐b:4,5‐b′]dithiophene‐2,6‐diyl‐alt‐3‐fluoro‐2‐[(2ethylhexyl) carbonyl]thieno[3,4‐b]thiophene‐4,6‐diyl] (PTB7) was used in CsPbI_3_ PQDSCs and it exhibited efficient electron blocking and hole extraction capability as well as the surface passivation effect, which may be attributed to the sulfur and carbonyl groups,^[^ ^108^ ^]^ thus conferring devices with excellent PCE and reasonable environmental stability.^[^ ^109^ ^]^ In addition, inorganic Cu_12_Sb_4_S_13_ QDs were also employed as HTMs in CsPbI_3_ PQDSCs,^[^ ^110^ ^]^ which possess lattice match with CsPbI_3_ PQDs. Not only do they have the advantages of suitable valence band energy level, high hole mobility, and nontoxicity, but also, they are beneficial for the hole extraction.^[^ ^111^ ^]^ Resultingly, the all‐inorganic PQDSCs achieve a PCE of 10.0% with higher *J*_sc_ (18.28 mA cm^−2^) and longer‐term stability.

## 3 PQDs Applied in Various Solar Cells

Apart from being main absorbing layer, PQDs are also employed to modify kinds of solar cells owing to their high PLQY and low excitation energies,^[^ ^112^ ^]^ that effectively increases the device performance as summarized in **Table** **2**. Concretely, the PQD films are able to be utilized as photo conversion layer and interface layer to enhance the performance of the photovoltaics. Additionally, PQDs are introduced as additive in organic solar cells (OSCs) and antisolvents forming perovskite films to achieve excellent properties for PSCs. These roles of PQDs are presented in detail in the following parts.

**Table 2:** Summary of the PQDs modifying various solar cells

Composition of active layer	PQDs for modification	Improvement of PCE [%]	Ref.
MAPbI_3_	CsPbBr_3_	14.7→16.4 19.7→20.8	^[^ ^113^ ^]^
MAPbI_3_	CsPbCl_3_	18.0→18.6	^[^ ^114^ ^]^
J71:ITIC		10.9→11.2	
c‐Si		17.5→18.0	
c‐Si	CsPbCl_1.5_Br_1.5_	18.1→21.5	^[^ ^115^ ^]^
c‐Si	CsPbCl_3_	19.6→20.7	^[^ ^116^ ^]^
c‐Si	MAPbBr_3_	16.1→16.4	^[^ ^117^ ^]^
		16.7→17.2	
CsPbI_2_Br	CsPbI_3_	13.5→14.5	^[^ ^118^ ^]^
MA_0.17_FA_0.83_Pb(I_0.83_Br_0.17_)_3_	CsPbI_3_	15.2→18.6	^[^ ^119^ ^]^
Cs_0.05_(FA_0.85_MA_0.15_)_0.95_Pb(I_0.85_Br_0.15_)_3_	CsPbI_1.15_Br_1.85_	19.5→21.1	^[^ ^120^ ^]^
MAPbI_3_	CsPbI_1.85_Br_1.15_	16.0→21.1	^[^ ^121^ ^]^
MAPbBr_3_	MAPbI_2.1_Br_0.9_	10.3→13.3	^[^ ^122^ ^]^
FAPbI_3_	Cs_0.57_FA_0.43_PbI_3_	19.6→20.8	^[^ ^123^ ^]^
FAMAPbI_x_Br_1‐x_	Cs_0.05_(MA_0.17_FA_0.83_)_0.95_ PbBr_3_	19.5→21.1	^[^ ^124^ ^]^
CsPbBr_3_	CsSnIBr_2_	5.5→9.1	^[^ ^125^ ^]^
CsPbBr_3_	CsMBr_3_ (M = Sn, Bi, Cu)	9.2→10.6	^[^ ^126^ ^]^
Sb_2_(S,Se)_3_	MAPbBr_3_	18.0→19.1	^[^ ^127^ ^]^
	CsPbBr_3_	18.0→21.5	
PbSe	CsPbBr_3_	4.8→7.2	^[^ ^128^ ^]^
PTB7‐Th:PC71BM	CsPbI_3_	7.9→10.8	^[^ ^129^ ^]^
PTB7‐Th/FOIC	CsPbI_3_	11.6→13.2	^[^ ^130^ ^]^
PBDB‐T:IT‐M	CsPbI_3_	10.7→11.6	^[^ ^131^ ^]^
FA_0.85_MA_0.15_Pb(I_0.85_Br_0.15_)_3_	CsPbBr_3_	18.0→19.5	^[^ ^132^ ^]^
MAPbI_3_	CsPbBr_3_	18.5→20.5	^[^ ^133^ ^]^
CsFAMAPbI*_x_* Br_1−_ *_x_*	CsPbBr_3_	19.1→20.0	^[^ ^134^ ^]^
MAPbI_3_	CsPbCl_2_Br	18.3→21.5	^[^ ^135^ ^]^
CsFAMAPbI*_x_* Br_1−_ *_x_*	CsPbBr_3_	19.1→21.0	^[^ ^136^ ^]^
	MAPbBr_3_	19.1→19.8	

### 3.1 Photo Conversion Layer

**Figure 7:**
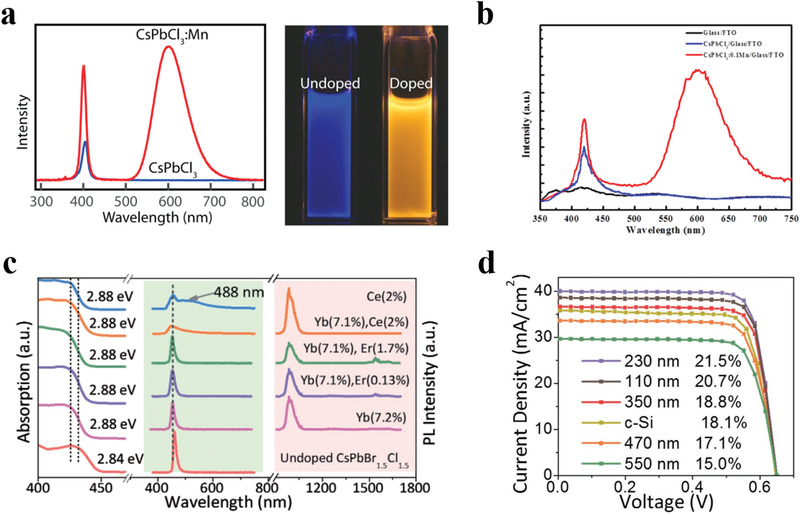
a) Photoluminescence of Mn‐doped and undoped CsPbCl_3_ PQDs and photographs of the sample under UV excitation. Reproduced with permission.^[^ ^137^ ^]^ Copyright 2016, American Chemical Society. b) Emission spectra (excited by 365 nm) of glass/FTO, CsPbCl_3_/glass/FTO, and CsPbCl_3_:0.1Mn/glass/FTO. Reproduced with permission.^[^ ^114^ ^]^ Copyright 2017, American Chemical Society. c) Absorption (left) and emission spectra (middle and right) of CsPbCl_1.5_Br_1.5_ PQDs codoping with various ions. d) *J*–*V* curves of the best SSCs coated with different thickness of perovskite film. Reproduced with permission.^[^ ^115^ ^]^ Copyright 2017, Wiley‐VCH.

Photon energy conversion is proposed as a universal strategy to further increasing the photovoltaic performance for solar cells. In general, the photo energy converters can convert multiple in‐active low‐energy photons into active photons to extend the absorption region or convert one high‐energy photons with radiation damage to multiple absorbable photons to increase quantum efficiency and photostability. The tunable photoluminescence outputs, high PLQYs and large Stokes shift to reduce the self‐reabsorption losses enable CsPbX_3_ PQDs with a wide band gap to be ideal photon energy converters as it can convert ultraviolet photons into visible photons. Chen et al. inserted CsPbBr_3_ PQDs layers between ETLs and vapor‐processed MAPbI_3_ to enhance PCE and stability for normal‐structure PSCs.^[^ ^113^ ^]^ On one hand, CsPbBr_3_ PQDs and hybrid perovskites film form a heterojunction, which can reduce defect density and boost electron transfer dynamics. On the other hand, they can convert the UV light into visible light by down‐conversion effect, so the modified devices showed higher external quantum efficiency in the short‐wavelength (300−500 nm) and retarded UV photo‐induced degradation. In addition, extrinsic ions doping into PQDs was demonstrated possessing various functions involving extending the Stokes shift, inhibiting self‐reabsorption loss, achieving broadband emission, enhancing PLQY and improving structural stability. With this in mind, Liu's group exhibited that Mn^2+^ doped CsPbCl_3_ PQDs (CsPbCl_3_:Mn) with large Stokes shift (higher than 200 nm) and yellow emission (**Figure** **7**a,b) can be applied onto the MAPbI_3_ perovskites, organic, and silicon solar cells.^[^ ^114^ ^]^ As the CsPbCl_3_:Mn PQDs effectively converting the normally wasted energy in the UV region (300−400 nm) into valuable visible light at ≈590 nm, both the PCE (especially the *J*_SC_) and photo‐stability of devices were improved. In addition, Song's group sequentially prepared CsPbCl_1.5_Br_1.5_:Yb^3+^, Ce^3+^ PQDs^[^ ^115^ ^]^ and CsPbCl_3_:Mn^2+^ PQDs,^[^ ^116^ ^]^ both achieving the photo energy conversion from ultraviolet‐visible region to visible‐near‐infrared region. As an example, CsPbCl_1.5_Br_1.5_:Yb^3+^, Ce^3+^ PQDs with large Strokes shift offer a high PLQY approaching to 146% and strong infrared emissions benefitting from efficient quantum cutting emission process (Figure 7c). Upon assembling CsPbCl_1.5_Br_1.5_:Yb^3+^, Ce^3+^ PQDs in front, the PCE of commercial crystalline‐silicon solar cells was enhanced from 18.1% to 21.5%, contributed by the increment in *J*_SC_ (Figure 7d).^[^ ^115^ ^]^

Besides, since PQDs uniformly embedded in a polymer matrix can efficiently overcome the issue of irreversible nanoparticle aggregation and undesirable luminescence quenching, MAPbBr_3_ PQDs/polyacrylonitrile (PAN) composite film was fabricated to improve the PCE of commercial silicon solar cells.^[^ ^117^ ^]^ With an unparalleled level of tunability of absorption and emission spectra due to the matched refractive index, MAPbBr_3_ PQDs together with PAN can convert the higher energy photons that cannot be sufficiently utilized into lower energy photons that can be well used for photocurrent generation. The easy and low‐cost fabrication based on MAPbBr_3_ PQDs makes it a practical way to achieve photovoltaic enhancement of c‐Si solar cells.

### 3.2 Interface Layer Enhancing Charge Dynamics

A multitude of studies have demonstrated the interface engineering is crucial for all kinds of solar cell with the purpose of facilitating the charge injection/separation efficiencies greatly, and substantially improving the device performance.^[^ ^138^ ^]^ On account of inorganic PQDs with tunable band position and orthogonal solvent feature,^[^ ^34^ ^]^ PQDs are regarded as an appropriate material to optimize interfaces of various solar cells

#### 3.2.1 Optimizing the Perovskite/HTL Interface for PSCs

Till now, CsPbI_3_ PQDs,^[^ ^118^, ^119^ ^]^ CsPbBr_3_ PQDs,^[^ ^132^ ^]^ CsPbI*_x_* Br_3−_ *_x_* PQDs,^[^ ^120^, ^121^ ^]^ MAPbI_2.1_Br_0.9_ PQDs,^[^ ^122^ ^]^ Cs_0.57_FA_0.43_PbI_3_ PQDs,^[^ ^123^ ^]^ Cs_0.05_(MA_0.17_FA_0.83_)_0.95_PbBr_3_ PQDs^[^ ^124^ ^]^ were introduced between the perovskite layers and the HTLs as interface layers, markedly enhancing the PCE and stability of PSCs. By virtue of their multifunctional properties,^[^ ^139^ ^]^ the key reason was revealed.^[^ ^140^ ^]^ It has been turned out PQD interlayers provide the multifunctional functions on the perovskite/HTL interface as follows: i) passivating the perovskites surface to reduce trap states probably contributed by OAm ligand; ii) promoting holes extraction from perovskites to HTLs by forming cascade energy levels; iii) improving hole mobilities of HTLs by regulating their polymer/molecule orientation.

Besides, certain PQDs also exhibited special optimization mechanism while applied in corresponding PSCs. For example, CsPbI_1.85_Br_1.15_ PQDs was used between perovskite and kinds of dopant‐free HTL interfaces which was also extended to large‐area solar modules that the solar module presented an obvious upgrade in *V*_OC_ and *FF* with the PQD interlayer and 17.6% efficiency was achieved at an 18.0 cm^2^ active area.^[^ ^121^ ^]^ Moreover, Cs_0.57_FA_0.43_PbI_3_ PQDs were deposited on the surface of Cs‐lean FAPbI_3_ perovskite films to circumvent the issue that only a limited amount of Cs can be alloyed owing to the intrinsically low solid‐solubility of Cs.^[^ ^123^ ^]^ The PQDs modification not only improves the charge dynamics in the devices but also significantly enhances the ambient stability. Also, our group successfully prepared CsPbI_3_ PQD layers in CsPbI_2_Br‐based PSCs forming a bilayer device,^[^ ^118^ ^]^ which expands the absorption threshold from 650 to 700 nm. Specially, I^−^ ions from the CsPbI_3_ PQDs and Br^−^ ions in the CsPbI_2_Br layer can exchange with each other throughout the bilayer interface, making the ideal bilayer CsPbI_2_Br/CsPbI_3_ PQD circuit change into a graded structure consisting of different CsPbI_2+_ *_x_* Br_1−_ *_x_* layers (0 ≤ *x* ≤ 1). The approach allows the potential to optimize the energy band alignment at the interface of the bilayer, leading to favorable band bending and improved carrier transport. The obtained PCE as high as 14.4% exhibit CsPbI_3_ PQD modification is a proper approach for improving the performance of inorganic PSCs.

#### 3.2.2 Optimizing Absorbers/Electrode Interface

PQDs are able to set a bridging energy level upon interface between perovskites and electrodes, functioning as mitigating energy barrier, reducing trap state density and hence suppressing interface recombination. For instance, Tang et al. introduced CsSnI_x_Br_3‐x_ PQDs into CsPbBr_3_/carbon electrodes interface to moderate the mismatch between the valence band of CsPbBr_3_ (−5.7 eV) and work function of carbon electrodes (−5.0 eV).^[^ ^125^ ^]^ Due to the intrinsically created tin vacancies,^[^ ^141^ ^]^ the p‐type CsSnBr_3−_ *_x_* I*_x_* extracted the photogenerated holes in CsPbBr_3_ layer and subsequently delivered to carbon electrodes. With the interface layers, all‐inorganic CsPbBr_3_ PSCs display an PCE of 9.1%, 66.3% higher in comparison to PQD‐free devices.

In further, PQDs were clearly proposed to be employed as HTLs in CsPbBr_3_ and Sb_2_(S,Se)_3_ solar cells, especially Br‐based PQDs involving CsMBr_3_ (M = Pb, Sn, Bi, Cu), and MAPbBr_3_ PQDs^[^ ^126^, ^127^ ^]^ due to their excellent stability ensured by the suitable tolerance factor. The PQDs generally lead to better‐matched energy level alignment and thus higher efficiency. Notably, the PQD‐based HTLs essentially confers superior device stability to PSCs in comparison with the conventional organic HTLs. Moreover, CsPbBr_3_ PQDs were dispersed in hexane and directly put onto the top of PbSe thin film using a machine dip‐coating method.^[^ ^128^ ^]^ Applying CsPbBr_3_ PQDs layer reduced the number of defect trapping states and secondary electron blocking effect, thus suppressing recombination at the interface between PbSe and Au. Also, the relatively small difference (0.15 eV) in the Fermi level‐valance band maximum separations between PbSe‐3‐mercaptopropoionic and CsPbBr_3_, and their large difference in *E*_g_ (1.30 to 2.56 eV) make it possible for the CsPbBr_3_ PQDs to be an electron block layer material with slight barrier extracting holes from the PbSe QD film. For these reasons, the simple way achieved the first reported PbSe QDSC (PCE over 7% with an FF over 60%).

### 3.3 Additive in OSCs

PQDs were extended to be additives in bulk heterojunction OSCs forming ternary components to further enhance the performance. In turn, the strong interaction between active materials (Lewis base) of OSCs and the Pb ions (Lewis acid) can also stabilize the PQD.^[^ ^130^ ^]^ The functions of PQDs in OSCs are summarized as follows. i) They usually act as a mediator in acceptor phase, such as [6,6]‐phenyl C_71_ butyric acid methyl‐ester (PC_71_BM), forming cascade band structure to favor exciton dissociation.^[^ ^129^ ^]^ ii) Highly crystalline PQDs could promote organic molecular ordering in active layers and in turn, thus benefiting charge transport process. iii) Their high dielectric‐constant and strong fluorescence constituent can suppress charge recombination by screening the coulombic interactions within the active layer and reduce voltage loss by enhancing external quantum efficiency (EQE) of electroluminescence (EL) of organic active layer. iv) High extinction coefficient and light scattering capability of the PQDs can enhance the light absorption of active layers.

As a typical example, Li et al. altered CsPbI_3_ PQDs by controlling size and doping heterovalent Bi^3+^, to effectively tune the energy level alignment of PQDs and OSC materials, thereby facilitating charge transfer between PQDs and OSC materials and leading to an increment in *V*_OC_.^[^ ^131^ ^]^ In addition, the incorporation of PQDs could lead to a higher FF by virtue of effectively modifying the nanoscale bulk heterojunction morphology toward more efficient charge collection. Through Rayleigh scattering and light absorption of the PQDs, the *J*_SC_ of the devices can also be improved. Finally, the CsPbI_3_ PQDs doped by 10 nm Bi raised the PCE of poly[(2,6‐(4,8‐bis(5‐(2‐ethylhexyl)thiophen‐2‐yl)‐benzo[1,2‐b:4,5‐b0]dithiophene))‐alt‐(5,5‐(10,30‐di‐2‐thienyl‐50,70‐bis(2‐ethylhexyl)benzo[10,20‐c:40,50‐c0]dithiophene‐4,8‐dione))] (PBDB‐T):3,9‐bis(6‐methyl‐2‐methylene‐ (3‐(1,1‐dicyanomethylene)‐indanone))‐5,5,11,11‐tetrakis(4‐hexylphenyl)‐dithieno[2,3‐d:2′,3‐d′]‐s‐indaceno[1,2‐b:5,6‐b′]dithiophene (IT‐M) based OSCs to 11.6%.

### 3.4 Additive in Antisolvents

Antisolvent treatment strategy is almost the most successful techniques to obtain perovskite films with high quality, which increases the nucleus density during film formation in order to fabricate pinhole‐free and uniform perovskite film, thereby facilitating improved PCE and stability. The incorporation of additives through antisolvents can simultaneously regulate the grain growth and charge transport of perovskite films, reducing the nonradiative carrier recombination and passivating both surface and grain boundary defects in situ.^[^ ^142^ ^]^ PQDs can be used as additives in antisolvent since they can deliver elements and surface capping molecular in perovskite layers, then achieving appropriate band alignment to facilitate the carrier extraction and reducing recombination loss and trap density.

CsPbBr_3_ PQDs with near‐ideal energy‐level alignment and excellent thermal stability had been successfully employed into chlorobenzene antisolvent to improve the bulk perovskite film quality and induced a passivation layer on the perovskite layer, retarding light‐induced degradation and enhancing the performance and stability of devices.^[^ ^132^, ^133^ ^]^ Accordingly, CsPbBr_3_ PQD modification exhibits significant potential to fabricate PSCs with rosy performance. Nevertheless, as we mentioned above, the surface‐passivating ligands with long alkyl chain in PQDs are not conductive to charge transport because of their extremely poor electrical conductivity.^[^ ^143^ ^]^ In order to solve the problem, Tang et al. employed a conductive diammonium porphyrin (ZnPy‐NH_3_Br) to displace the long‐chain organic ligands in CsPbBr_3_ PQDs coated on a CsMAFA film.^[^ ^134^ ^]^ With porphyrin‐bridging, a stable 0D–2D perovskite capping layer were formed. Ultimately, compared with pure perovskite and CsPbBr_3_ PQDs modification, the PCE is higher with CsPbBr_3_ PQDs modified treated by ZnPy‐NH_3_Br. The bulk perovskite presents more durable stability after ligand‐exchange, too. The multi‐junction stacking technique has been proved effective for various solar cells, which has also been adopted for the inorganic perovskite cells.^[^ ^144^ ^]^

**Figure 8:**
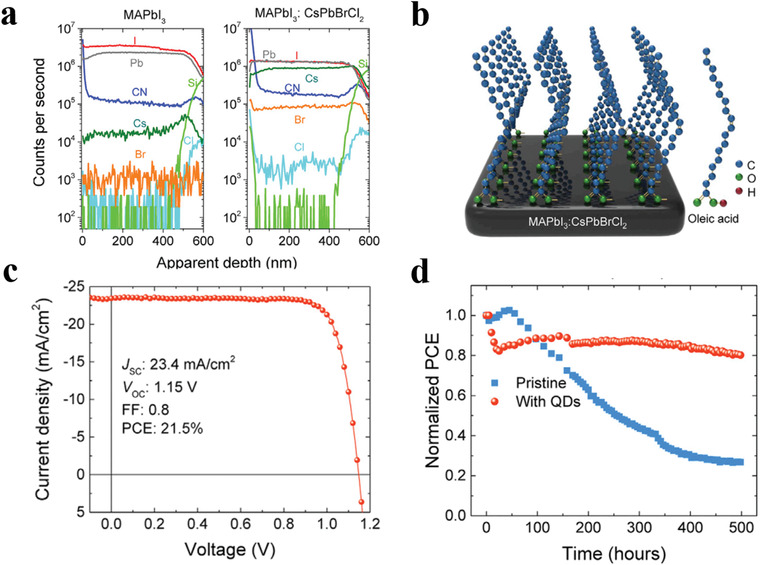
a) Depth‐dependent elemental distribution for films (the pristine MAPbI_3_ and the film with 0.25 wt% of PQDs in anti‐solvent). b) Schematic diagram of the uniform distribution of elements across the MAPbI_3_ film and self‐assembly of the OA molecules on the surface of the MAPbI_3_ film. c) *J–V* characteristic of the champion device with 0.25 wt% of PQDs in anti‐solvent. d) PCE for the encapsulated device without PQDs and with 0.25 wt% PQDs over time. Reproduced with permission.^[^ ^135^ ^]^ Copyright 2019, Elsevier Inc.

In addition to CsPbBr_3_ PQDs, Zheng and his co‐workers reported a strategy that CsPbCl_2_Br PQDs coated by OA were put into antisolvent toluene.^[^ ^135^ ^]^ Specially, they utilized the PQDs to realize elemental dopants and anchor ligands across an MAPbI_3_ film (**Figure** **8**a,b). They pointed out that the surface ligand of the PQDs benefits the defect passivation of PSCs through reducing the film's trap state density with improved PCE of device (Figure 8c). Concurrently, the PQD‐modified PSCs possess superior stability with surface hydrophobic (Figure 8d).

On the latter, rarely, CsPbBr_3_ and MAPbBr_3_ PQDs were introduced into the inverted planar PSCs with triple‐cation perovskite films during the film‐formation process.^[^ ^136^ ^]^ Specially, adding CsPbBr_3_ PQDs with the size of ≈18 nm into anti‐solvent leads to an improved crystal quality of the bulk perovskite films, modifying the surface morphology, electronic properties and crystal structures. Furthermore, the devices exhibit a high *V*_OC_ of 1.19 V because the built‐in potentials determined by the quasi‐Fermi level spitting were considerably enhanced with the reduced non‐radiative recombination losses.

## 4 Conclusion and Perspective

In this review, the application of PQDs in photovoltaic devices were reviewed thoroughly. In the first place, the comprehensive advances of PQDSCs are summarized in terms of ligand engineering, additive engineering and hybrid composition engineering. Note that ligand engineering is a main and powerful strategy to tailor the properties of PQDs, making it even more charming for practical applications. These strategies confer PQD films with various properties including enhanced stability, reduced defect density and enhanced carrier transport capability, leading to excellent optoelectronic performance for PQDSCs. Also, the reasons behind the undesired performance of PQDSCs were analyzed in terms of photovoltaic parameters and carrier dynamics to provide researchers with some optimizing directions. In the second place, the various functions of PQDs in solar cells were reviewed including light conversion, interface modification and so on.

Despite the significant progress described above, the application of PQDs is only at its infancy and the highest PCE still cannot compete with the bulk perovskite‐based devices. A series of scientific issues should be resolved by combining experimental and theoretical methodologies. First, the density and variety of capping ligands are crucial since PQDs require certain density of ligands to maintain phase stability while PQDSCs entails removing ligands to ensure charge dynamics. This dilemma may be resolved by preparing PQDs with intrinsic stability. Moreover, compared with the developed bulk PSCs utilizing perovskites of 1.5–1.6 eV, highly efficient PQDSCs were mainly achieved by using >1.65 eV PQDs, limiting the further improvement of PCE. Thus the composition of PQDs should be further regulated to achieve smaller bandgap. Second, there are few studies verifying the long‐term operational stability of PQDSCs toward heat, light, and humidity, although researchers emphasized that PQDs can remain phase stability owing to the surface effect. Third, the methods for preparing PQDs are conducted in minitype glass instrument, for example, three‐neck‐flask, and the yield is in gram level. Thus, the synthesis and post‐treatment process should be advanced enabling the compatibility with large‐scale synthesis and continuous production of large‐area PQD films. In addition, the toxicity of Pb and cost must be taken into account in term of the industrial‐scale use of PQDSCs. For the former, Pb is harmful to the environment and human health, which should be replaced by green elements.^[^ ^145^ ^]^ For example, the environment‐friendly CsSnI_3_ PQDs with suitable bandgap possess greater potential for high PCE. For the latter, taking CsPbI_3_ PQDSCs for example, Jean et al. assessed the median synthesizing costs are $73 per g (≈$0.74 per W) by Monte Carlo simulations,^[^ ^146^ ^]^ which cost more than PbS QDs (0.15 to 0.84 $ per W) and silicon devices (≈$0.20–0.40 per W).^[^ ^147^ ^]^ Additionally, the cost of operating conditions, manufacturing, production equipment is relatively high. To occupy a space in the commercial market, higher PCE with longer lifetimes of PQDSCs would be required.

Undoubtedly, PQDs are promising to be “game changer” in the field of photovoltaics, which has displayed great potential in large‐scale manufacturing, low‐temperature flexible fabrication and semi‐transparent or tandem photovoltaic devices. Further development and implementation of these materials could potentially result in more effective solar energy harnessing technologies. Certainly, it poses a higher challenge to scientific researchers with the purpose of bringing out the potentials and advantages of PQDs.

## Conflict of Interest

The authors declare no conflict of interest.

## References

[1] K. Yamada , H. Kawaguchi , T. Matsui , T. Okuda , S. Ichiba , Bull. Chem. Soc. Jpn. 1990, 63, 2521.

[2] J. H. Noh , S. H. Im , J. H. Heo , T. N. Mandal , S. I. Seok , Nano Lett. 2013, 13, 1764. 23517331 10.1021/nl400349b

[3] a) G. Xing , N. Mathews , S. Sun , S. S. Lim , Y. M. Lam , M. Grätzel , S. Mhaisalkar , T. C. Sum , Science 2013, 342, 344; 24136965 10.1126/science.1243167

[4] A. Kojima , K. Teshima , Y. Shirai , T. Miyasaka , J. Am. Chem. Soc. 2009, 131, 6050. 19366264 10.1021/ja809598r

[5] Best research‐cell efficiency chart, http://www.nrel.gov/pv/cell‐efficiency.chart.jpg (accessed 26th July 2021).

[6] K. Yoshikawa , H. Kawasaki , W. Yoshida , T. Irie , K. Konishi , K. Nakano , T. Uto , D. Adachi , M. Kanematsu , H. Uzu , K. Yamamoto , Nat. Energy 2017, 2, 8.

[7] a) A. Miyata , A. Mitioglu , P. Plochocka , O. Portugall , J. T.‐W. Wang , S. D. Stranks , H. J. Snaith , R. J. Nicholas , Nature Phys. 2015, 11, 582;

[8] a) H. C. Wang , S. Y. Lin , A. C. Tang , B. P. Singh , H. C. Tong , C. Y. Chen , Y. C. Lee , T. L. Tsai , R. S. Liu , Angew. Chem., Int. Ed. 2016, 55, 7924; 10.1002/anie.201603698 27239980

[9] a) Y. Wang , T. Zhang , M. Kan , Y. Zhao , J. Am. Chem. Soc. 2018, 140, 12345; 30247030 10.1021/jacs.8b07927

[10] R. Wang , M. Mujahid , Y. Duan , Z. K. Wang , J. Xue , Y. Yang , Adv. Funct. Mater. 2019, 29, 1808843.

[11] a) C. Yi , J. Luo , S. Meloni , A. Boziki , N. Ashari‐Astani , C. Grätzel , S. M. Zakeeruddin , U. Röthlisberger , M. Grätzel , Energy Environ. Sci. 2016, 9, 656;

[12] M. Du , X. Zhu , L. Wang , H. Wang , J. Feng , X. Jiang , Y. Cao , Y. Sun , L. Duan , Y. Jiao , K. Wang , X. Ren , Z. Yan , S. Pang , S. F. Liu , Adv. Mater. 2020, 32, 2004979. 10.1002/adma.202004979 33079444

[13] J. H. Heo , F. Zhang , C. Xiao , S. J. Heo , J. K. Park , J. J. Berry , K. Zhu , S. H. Im , Joule 2021, 5, 481.

[14] J. Feng , Y. Jiao , H. Wang , X. Zhu , Y. Sun , M. Du , Y. Cao , D. Yang , S. Liu , Energy Environ. Sci. 2021, 14, 3035.

[15] a) J. Yuan , A. Hazarika , Q. Zhao , X. Ling , T. Moot , W. Ma , J. M. Luther , Joule 2020, 4, 1160;

[16] Y. Wang , X. Li , J. Song , L. Xiao , H. Zeng , H. Sun , Adv. Mater. 2015, 27, 7101. 26448638 10.1002/adma.201503573

[17] H. Chen , J. M. Pina , Y. Hou , E. H. Sargent , Adv. Energy Mater. 2021, 2100774.

[18] Q. A. Akkerman , G. Rainò , M. V. Kovalenko , L. Manna , Nat. Mater. 2018, 17, 394. 29459748 10.1038/s41563-018-0018-4

[19] a) A. Swarnkar , R. Chulliyil , V. K. Ravi , M. Irfanullah , A. Chowdhury , A. Nag , Angew. Chem., Int. Ed. 2015, 127, 15644; 10.1002/anie.201508276 26546495

[20] Q. A. Akkerman , V. D'Innocenzo , S. Accornero , A. Scarpellini , A. Petrozza , M. Prato , L. Manna , J. Am. Chem. Soc. 2015, 137, 10276. 26214734 10.1021/jacs.5b05602 PMC4543997

[21] C. de Weerd , L. Gomez , A. Capretti , D. M. Lebrun , E. Matsubara , J. Lin , M. Ashida , F. C. Spoor , L. D. Siebbeles , A. J. Houtepen , Nat. Commun. 2018, 9, 4199. 30305623 10.1038/s41467-018-06721-0 PMC6180104

[22] E. M. Sanehira , A. R. Marshall , J. A. Christians , S. P. Harvey , P. N. Ciesielski , L. M. Wheeler , P. Schulz , L. Y. Lin , M. C. Beard , J. M. Luther , Sci. Adv. 2017, 3, eaao4204. 29098184 10.1126/sciadv.aao4204 PMC5659658

[23] Z. Yang , M. Wang , J. Li , J. Dou , H. Qiu , J. Shao , ACS Appl. Mater. Interfaces 2018, 10, 26387. 30001101 10.1021/acsami.8b07334

[24] J. Yuan , C. Bi , S. Wang , R. Guo , T. Shen , L. Zhang , J. Tian , Adv. Funct. Mater. 2019, 29, 1906615.

[25] W. Cai , H. Li , M. Li , M. Wang , H. Wang , J. Chen , Z. Zang , J. Phys. D Appl. Phys. 2021, 54, 293002.

[26] H. He , T. C. Sum , Dalton Trans. 2020, 49, 15149. 33000825 10.1039/d0dt02538k

[27] X. Li , X. Gao , X. Zhang , X. Shen , M. Lu , J. Wu , Z. Shi , V. L. Colvin , J. Hu , X. Bai , Adv. Sci. 2021, 8, 2003334. 10.1002/advs.202003334 PMC7887601 33643803

[28] A. Swarnkar , A. R. Marshall , E. M. Sanehira , B. D. Chernomordik , D. T. Moore , J. A. Christians , T. Chakrabarti , J. M. Luther , Science 2016, 354, 92. 27846497 10.1126/science.aag2700

[29] Q. Zhao , A. Hazarika , X. Chen , S. P. Harvey , B. W. Larson , G. R. Teeter , J. Liu , T. Song , C. Xiao , L. Shaw , M. Zhang , G. Li , M. C. Beard , J. M. Luther , Nat. Commun. 2019, 10, 2842. 31253800 10.1038/s41467-019-10856-z PMC6599010

[30] L. Protesescu , S. Yakunin , M. I. Bodnarchuk , F. Krieg , R. Caputo , C. H. Hendon , R. X. Yang , A. Walsh , M. V. Kovalenko , Nano Lett. 2015, 15, 3692. 25633588 10.1021/nl5048779 PMC4462997

[31] F. Zhang , H. Zhong , C. Chen , X.‐g. Wu , X. Hu , H. Huang , J. Han , B. Zou , Y. Dong , ACS Nano 2015, 9, 4533. 25824283 10.1021/acsnano.5b01154

[32] I. Lignos , S. Stavrakis , G. Nedelcu , L. Protesescu , A. J. deMello , M. V. Kovalenko , Nano Lett. 2016, 16, 1869. 26836149 10.1021/acs.nanolett.5b04981

[33] X. Li , F. Cao , D. Yu , J. Chen , Z. Sun , Y. Shen , Y. Zhu , L. Wang , Y. Wei , Y. Wu , H. Zeng , Small 2017, 13, 1603996. 10.1002/smll.201603996 28067991

[34] G. Nedelcu , L. Protesescu , S. Yakunin , M. I. Bodnarchuk , M. J. Grotevent , M. V. Kovalenko , Nano Lett. 2015, 15, 5635. 26207728 10.1021/acs.nanolett.5b02404 PMC4538456

[35] Y. Zhang , T. D. Siegler , C. J. Thomas , M. K. Abney , T. Shah , A. De Gorostiza , R. M. Greene , B. A. Korgel , J. Chem. Mater. 2020, 32, 5410.

[36] Y. Bai , M. Hao , S. Ding , P. Chen , L. Wang , Adv. Mater. 2021, 2105958. 10.1002/adma.202105958 34643300

[37] J. Xue , J.‐W. Lee , Z. Dai , R. Wang , S. Nuryyeva , M. E. Liao , S.‐Y. Chang , L. Meng , D. Meng , P. Sun , O. Lin , M. S. Goorsky , Y. Yang , Joule 2018, 2, 1866.

[38] K. Chen , Q. Zhong , W. Chen , B. Sang , Y. Wang , T. Yang , Y. Liu , Y. Zhang , H. Zhang , Adv. Funct. Mater. 2019, 29, 1900991.

[39] J. Shi , F. Li , Y. Jin , C. Liu , B. Cohen‐Kleinstein , S. Yuan , Y. Li , Z. K. Wang , J. Yuan , W. Ma , Angew. Chem., Int. Ed. 2020, 132, 22414. 10.1002/anie.202010440 32840045

[40] S. Cho , J. Kim , S. M. Jeong , M. J. Ko , J.‐S. Lee , Y. Kim , Chem. Mater. 2020, 32, 8808.

[41] R. Han , Q. Zhao , J. Su , X. Zhou , X. Ye , X. Liang , J. Li , H. Cai , J. Ni , J. Zhang , J. Phys. Chem. C 2021, 125, 8469.

[42] J. Kim , S. Cho , F. Dinic , J. Choi , C. Choi , S. M. Jeong , J.‐S. Lee , O. Voznyy , M. J. Ko , Y. Kim , Nano Energy 2020, 75, 104985.

[43] a) X. Ling , J. Yuan , X. Zhang , Y. Qian , S. M. Zakeeruddin , B. W. Larson , Q. Zhao , J. Shi , J. Yang , K. Ji , Y. Zhang , Y. Wang , C. Zhang , S. Duhm , J. M. Luther , M. Gratzel , W. Ma , Adv. Mater. 2020, 32, 2001906; 10.1002/adma.202001906 32449221

[44] D. Jia , J. Chen , M. Yu , J. Liu , E. M. J. Johansson , A. Hagfeldt , X. Zhang , Small 2020, 16, 2001772. 10.1002/smll.202001772 32419275

[45] J. Khan , X. Zhang , J. Yuan , Y. Wang , G. Shi , R. Patterson , J. Shi , X. Ling , L. Hu , T. Wu , S. Dai , W. Ma , ACS Energy Lett. 2020, 5, 3322.

[46] J. Xue , R. Wang , L. Chen , S. Nuryyeva , T. H. Han , T. Huang , S. Tan , J. Zhu , M. Wang , Z. K. Wang , C. Zhang , J. W. Lee , Y. Yang , Adv. Mater. 2019, 31, 1900111. 10.1002/adma.201900111 31343086

[47] J. Yuan , X. Zhang , J. Sun , R. Patterson , H. Yao , D. Xue , Y. Wang , K. Ji , L. Hu , S. Huang , D. Chu , T. Wu , J. Hou , J. Yuan , Adv. Funct. Mater. 2021, 31, 2101272.

[48] Y. Wang , J. Yuan , X. Zhang , X. Ling , B. W. Larson , Q. Zhao , Y. Yang , Y. Shi , J. M. Luther , W. Ma , Adv. Mater. 2020, 32, 2000449. 10.1002/adma.202000449 32609406

[49] J. Shi , F. Li , J. Yuan , X. Ling , S. Zhou , Y. Qian , W. Ma , J. Mater. Chem. A 2019, 7, 20936.

[50] L. Zhang , C. Kang , G. Zhang , Z. Pan , Z. Huang , S. Xu , H. Rao , H. Liu , S. Wu , X. Wu , X. Li , Z. Zhu , X. Zhong , A. K. Y. Jen , Adv. Funct. Mater. 2020, 31, 2005930.

[51] C. Bi , X. Sun , X. Huang , S. Wang , J. Yuan , J. X. Wang , T. Pullerits , J. Tian , Chem. Mater. 2020, 32, 6105.

[52] D. Ghosh , M. Y. Ali , A. Ghosh , A. Mandal , S. Bhattacharyya , J. Phys. Chem. C 2021, 125, 5485.

[53] F. Liu , C. Ding , Y. Zhang , T. Kamisaka , Q. Zhao , J. M. Luther , T. Toyoda , S. Hayase , T. Minemoto , K. Yoshino , B. Zhang , S. Dai , J. Jiang , S. Tao , Q. Shen , Chem. Mater. 2019, 31, 798.

[54] Q. Wang , Z. Jin , D. Chen , D. Bai , H. Bian , J. Sun , G. Zhu , G. Wang , S. F. Liu , Adv. Energy Mater. 2018, 8, 1800007.

[55] L. Hu , Q. Zhao , S. Huang , J. Zheng , X. Guan , R. Patterson , J. Kim , L. Shi , C. H. Lin , Q. Lei , D. Chu , W. Tao , S. Cheong , R. D. Tilley , A. W. Y. Ho‐Baillie , J. M. Luther , J. Yuan , T. Wu , Nat. Commun. 2021, 12, 466. 33473106 10.1038/s41467-020-20749-1 PMC7817685

[56] D. Jia , J. Chen , X. Mei , W. Fan , S. Luo , M. Yu , J. Liu , X. Zhang , Energy Environ. Sci. 2021, 14, 4599.

[57] X. Zhang , H. Huang , X. Ling , J. Sun , X. Jiang , Y. Wang , D. Xue , L. Huang , L. Chi , J. Yuan , W. Ma , Adv. Mater. 2021, 10.1002/adma.202105977. 34695259

[58] a) D. Ghosh , M. Y. Ali , D. K. Chaudhary , S. Bhattacharyya , Sol. Energ. Mat. Sol. C. 2018, 185, 28;

[59] S. Y. Park , H. C. Shim , ACS Appl. Mater. Interfaces 2020, 12, 57124. 33289539 10.1021/acsami.0c17877

[60] M. Hao , Y. Bai , S. Zeiske , L. Ren , J. Liu , Y. Yuan , N. Zarrabi , N. Cheng , M. Ghasemi , P. Chen , M. Lyu , D. He , J.‐H. Yun , Y. Du , Y. Wang , S. Ding , A. Armin , P. Meredith , G. Liu , H.‐M. Cheng , L. Wang , Nat. Energy 2020, 5, 79.

[61] S. Christodoulou , F. Di Stasio , S. Pradhan , A. Stavrinadis , G. Konstantatos , J. Phys. Chem. C 2018, 122, 7621.

[62] F. Liu , C. Ding , Y. Zhang , T. S. Ripolles , T. Kamisaka , T. Toyoda , S. Hayase , T. Minemoto , K. Yoshino , S. Dai , M. Yanagida , H. Noguchi , Q. Shen , J. Am. Chem. Soc. 2017, 139, 16708. 29091445 10.1021/jacs.7b08628

[63] a) D. Zhang , S. W. Eaton , Y. Yu , L. Dou , P. Yang , J. Am. Chem. Soc. 2015, 137, 9230; 26181343 10.1021/jacs.5b05404

[64] Y. Zhao , K. Zhu , J. Phys. Chem. Lett. 2013, 4, 2880.

[65] Y. Kim , E. Yassitepe , O. Voznyy , R. Comin , G. Walters , X. Gong , P. Kanjanaboos , A. F. Nogueira , E. H. Sargent , ACS Appl. Mater. Interfaces 2015, 7, 25007. 26529572 10.1021/acsami.5b09084

[66] J. B. Hoffman , G. Zaiats , I. Wappes , P. V. Kamat , J. Chem. Mater. 2017, 29, 9767.

[67] H. Huang , F. Zhao , L. Liu , F. Zhang , X.‐g. Wu , L. Shi , B. Zou , Q. Pei , H. Zhong , ACS Appl. Mater. Interfaces 2015, 7, 28128. 26652661 10.1021/acsami.5b10373

[68] Q. Shang , B. D. Piercy , M. D. Losego , T. Lian , J. Phys. Chem. C 2019, 123, 21415.

[69] H. Zhu , K. Miyata , Y. Fu , J. Wang , P. P. Joshi , D. Niesner , K. W. Williams , S. Jin , X.‐Y. Zhu , Science 2016, 353, 1409. 27708033 10.1126/science.aaf9570

[70] G. E. Eperon , S. D. Stranks , C. Menelaou , M. B. Johnston , L. M. Herz , H. J. Snaith , Energy Environ. Sci. 2014, 7, 982.

[71] H. Huang , M. I. Bodnarchuk , S. V. Kershaw , M. V. Kovalenko , A. L. Rogach , ACS Energy Lett. 2017, 2, 2071. 28920080 10.1021/acsenergylett.7b00547 PMC5594444

[72] a) M. Kulbak , S. Gupta , N. Kedem , I. Levine , T. Bendikov , G. Hodes , D. Cahen , J. Phys. Chem. Lett. 2016, 7, 167; 26700466 10.1021/acs.jpclett.5b02597

[73] a) J. Dai , J. Xi , L. Li , J. Zhao , Y. Shi , W. Zhang , C. Ran , B. Jiao , X. Hou , X. Duan , Z. Wu , Angew Chem., Int. Ed. 2018, 57, 5754; 10.1002/anie.201801780 29573090

[74] C. Bi , S. V. Kershaw , A. L. Rogach , J. Tian , Adv. Funct. Mater. 2019, 29, 1902446.

[75] L. M. Wheeler , E. M. Sanehira , A. R. Marshall , P. Schulz , M. Suri , N. C. Anderson , J. A. Christians , D. Nordlund , D. Sokaras , T. Kroll , S. P. Harvey , J. J. Berry , L. Y. Lin , J. M. Luther , J. Am. Chem. Soc. 2018, 140, 10504. 30044630 10.1021/jacs.8b04984

[76] a) J. De Roo , M. Ibanez , P. Geiregat , G. Nedelcu , W. Walravens , J. Maes , J. C. Martins , I. Van Driessche , M. V. Kovalenko , Z. Hens , ACS Nano 2016, 10, 2071; 26786064 10.1021/acsnano.5b06295

[77] R. Azmi , S. Sinaga , H. Aqoma , G. Seo , T. K. Ahn , M. Park , S.‐Y. Ju , J.‐W. Lee , T.‐W. Kim , S.‐H. Oh , Nano Energy 2017, 39, 86.

[78] J. Kim , B. Koo , W. H. Kim , J. Choi , C. Choi , S. J. Lim , J.‐S. Lee , D.‐H. Kim , M. J. Ko , Y. Kim , Nano Energy 2019, 66, 104130.

[79] X. Ling , S. Zhou , J. Yuan , J. Shi , Y. Qian , B. W. Larson , Q. Zhao , C. Qin , F. Li , G. Shi , C. Stewart , J. Hu , X. Zhang , J. M. Luther , S. Duhm , W. Ma , Adv. Energy Mater. 2019, 9, 1900721.

[80] S. B. Shivarudraiah , M. Ng , C. H. A. Li , J. E. Halpert , ACS App. Energy Mater. 2020, 3, 5620.

[81] a) R. Yuan , L. Shen , C. Shen , J. Liu , L. Zhou , W. Xiang , X. Liang , Chem. Commun. 2018, 54, 3395; 10.1039/C8CC00243F 29553148

[82] Q. Chen , L. Chen , F. Ye , T. Zhao , F. Tang , A. Rajagopal , Z. Jiang , S. Jiang , A. K.‐Y. Jen , Y. Xie , Nano Lett. 2017, 17, 3231. 28337916 10.1021/acs.nanolett.7b00847

[83] J. Kim , S. Cho , F. Dinic , J. Choi , C. Choi , S. M. Jeong , J.‐S. Lee , O. Voznyy , M. J. Ko , Y. Kim , Nano Energy 2021, 85, 106017.

[84] C. Lin , S. Li , W. Zhang , C. Shao , Z. Yang , ACS Appl. Energy Mater. 2018, 1, 1374.

[85] M. V. Kovalenko , L. Protesescu , M. I. Bodnarchuk , Science 2017, 358, 745. 29123061 10.1126/science.aam7093

[86] I. Lignos , V. Morad , Y. Shynkarenko , C. Bernasconi , R. M. Maceiczyk , L. Protesescu , F. Bertolotti , S. Kumar , S. T. Ochsenbein , N. Masciocchi , A. Guagliardi , C.‐J. Shih , M. I. Bodnarchuk , A. J. deMello , M. V. Kovalenko , ACS Nano 2018, 12, 5504. 29754493 10.1021/acsnano.8b01122 PMC6024237

[87] M. Suri , A. Hazarika , B. W. Larson , Q. Zhao , M. Vallés‐Pelarda , T. D. Siegler , M. K. Abney , A. J. Ferguson , B. A. Korgel , J. M. Luther , ACS Energy Lett. 2019, 4, 1954.

[88] D. J. Kubicki , D. Prochowicz , A. Hofstetter , P. Péchy , S. M. Zakeeruddin , M. Grätzel , L. Emsley , J. Am. Chem. Soc. 2017, 139, 10055. 28641413 10.1021/jacs.7b04930

[89] a) A. F. Gualdrón‐Reyes , S. J. Yoon , E. M. Barea , S. Agouram , V. Muñoz‐Sanjosé , Á. M. Meléndez , M. E. Niño‐Gómez , I. Mora‐Seró , ACS Energy Lett. 2019, 4, 54; 30662954 10.1021/acsenergylett.8b02207 PMC6333216

[90] a) Y. Wang , M. I. Dar , L. K. Ono , T. Zhang , M. Kan , Y. Li , L. Zhang , X. Wang , Y. Yang , X. Gao , Science 2019, 365, 591; 31395783 10.1126/science.aav8680

[91] H. Utzat , W. Sun , A. E. Kaplan , F. Krieg , M. Ginterseder , B. Spokoyny , N. D. Klein , K. E. Shulenberger , C. F. Perkinson , M. V. Kovalenko , Science 2019, 363, 1068. 30792359 10.1126/science.aau7392

[92] a) H. Cho , S.‐H. Jeong , M.‐H. Park , Y.‐H. Kim , C. Wolf , C.‐L. Lee , J. H. Heo , A. Sadhanala , N. Myoung , S. Yoo , Science 2015, 350, 1222; 26785482 10.1126/science.aad1818

[93] S. Mahesh , J. M. Ball , R. D. J. Oliver , D. P. McMeekin , P. K. Nayak , M. B. Johnston , H. J. Snaith , Energy. Environ. Sci. 2020, 13, 258.

[94] a) J. Liu , K. Xian , L. Ye , Z. Zhou , Adv. Mater. 2021, 33, 2008115; 10.1002/adma.202008115 34085736

[95] N. Wu , Y. Wu , D. Walter , H. Shen , T. Duong , D. Grant , C. Barugkin , X. Fu , J. Peng , T. White , K. Catchpole , K. Weber , Energy Technol. 2017, 5, 1827.

[96] J. Yuan , C. Bi , J. Xi , R. Guo , J. Tian , J. Phys. Chem. Lett. 2021, 12, 1018. 33470817 10.1021/acs.jpclett.0c03628

[97] H. C. Woo , J. W. Choi , J. Shin , S.‐H. Chin , M. H. Ann , C.‐L. Lee , J. Phys. Chem. Lett. 2018, 9, 4066. 29975057 10.1021/acs.jpclett.8b01593

[98] J. Wang , I. Mora‐Seró , Z. Pan , K. Zhao , H. Zhang , Y. Feng , G. Yang , X. Zhong , J. Bisquert , J. Am. Chem. Soc. 2013, 135, 15913. 24070636 10.1021/ja4079804

[99] F. Li , S. Zhou , J. Yuan , C. Qin , Y. Yang , J. Shi , X. Ling , Y. Li , W. Ma , ACS Energy Lett. 2019, 4, 2571.

[100] a) M. M. Lee , J. Teuscher , T. Miyasaka , T. N. Murakami , H. J. Snaith , Science 2012, 338, 643; 23042296 10.1126/science.1228604

[101] K. Ji , J. Yuan , F. Li , Y. Shi , X. Ling , X. Zhang , Y. Zhang , H. Lu , J. Yuan , W. Ma , J. Mater. Chem. A 2020, 8, 8104.

[102] F. Liu , Y. Zhang , C. Ding , T. Toyoda , Y. Ogomi , T. S. Ripolles , S. Hayase , T. Minemoto , K. Yoshino , S. Dai , Q. Shen , J. Phys. Chem. Lett. 2018, 9, 294. 29286666 10.1021/acs.jpclett.7b03062

[103] K. Chen , W. Jin , Y. Zhang , T. Yang , P. Reiss , Q. Zhong , U. Bach , Q. Li , Y. Wang , H. Zhang , J. Am. Chem. Soc. 2020, 142, 3775. 31967471 10.1021/jacs.9b10700

[104] C. Ding , F. Liu , Y. Zhang , D. Hirotani , X. Rin , S. Hayase , T. Minemoto , T. Masuda , R. Wang , Q. Shen , Nano Energy 2020, 67, 104267.

[105] D. Stockwell , Y. Yang , J. Huang , C. Anfuso , Z. Huang , T. Lian , J. Phys. Chem. C 2010, 114, 6560.

[106] Q. Zhang , C. S. Dandeneau , X. Zhou , G. Cao , Adv. Mater. 2009, 21, 4087.

[107] S. Lim , J. Kim , J. Y. Park , J. Min , S. Yun , T. Park , Y. Kim , J. Choi , ACS Appl. Mater. Interfaces 2021, 13, 6119. 33499586 10.1021/acsami.0c15484

[108] a) Q. Zeng , X. Zhang , X. Feng , S. Lu , Z. Chen , X. Yong , S. A. T. Redfern , H. Wei , H. Wang , H. Shen , W. Zhang , W. Zheng , H. Zhang , J. S. Tse , B. Yang , Adv. Mater. 2018, 30, 1705393; 10.1002/adma.201705393 29333763

[109] a) B. Carsten , J. M. Szarko , H. J. Son , W. Wang , L. Lu , F. He , B. S. Rolczynski , S. J. Lou , L. X. Chen , L. Yu , J. Am. Chem. Soc. 2011, 133, 20468; 22077184 10.1021/ja208642b

[110] Y. Liu , X. Zhao , Z. Yang , Q. Li , W. Wei , B. Hu , W. Chen , ACS Appl. Energy Mater. 2020, 3, 3521.

[111] a) J. Van Embden , K. Latham , N. W. Duffy , Y. Tachibana , J. Am. Chem. Soc. 2013, 135, 11562; 23876109 10.1021/ja402702x

[112] Y. Tian , A. Merdasa , M. Peter , M. Abdellah , K. Zheng , C. S. Ponseca Jr , T. N. Pullerits , A. Yartsev , V. Sundström , I. G. Scheblykin , Nano Lett. 2015, 15, 1603; 25706329 10.1021/nl5041397

[113] C. Chen , Y. Wu , L. Liu , Y. Gao , X. Chen , W. Bi , X. Chen , D. Liu , Q. Dai , H. Song , Adv. Sci. 2019, 6, 1802046. 10.1002/advs.201802046 PMC6548969 31179207

[114] Q. Wang , X. Zhang , Z. Jin , J. Zhang , Z. Gao , Y. Li , S. F. Liu , ACS Energy Lett. 2017, 2, 1479.

[115] D. Zhou , D. Liu , G. Pan , X. Chen , D. Li , W. Xu , X. Bai , H. Song , Adv. Mater. 2017, 29, 1704149. 10.1002/adma.201704149 28961346

[116] R. Sun , D. Zhou , Y. Wang , W. Xu , N. Ding , L. Zi , X. Zhuang , X. Bai , H. Song , Nanoscale 2020, 12, 18621. 32970067 10.1039/d0nr04885b

[117] L. Meng , X.‐G. Wu , S. Ma , L. Shi , M. Zhang , L. Wang , Y. Chen , Q. Chen , H. Zhong , Nanophotonics 2019, 9, 93.

[118] H. Bian , D. Bai , Z. Jin , K. Wang , L. Liang , H. Wang , J. Zhang , Q. Wang , S. Liu , Joule 2018, 2, 1500.

[119] C. Liu , M. Hu , X. Zhou , J. Wu , L. Zhang , W. Kong , X. Li , X. Zhao , S. Dai , B. Xu , C. Cheng , NPG Asia Mater. 2018, 10, 552.

[120] S. Akin , Y. Altintas , E. Mutlugun , S. Sonmezoglu , Nano Energy 2019, 60, 557.

[121] F. Cheng , R. He , S. Nie , C. Zhang , J. Yin , J. Li , N. Zheng , B. Wu , J. Am. Chem. Soc. 2021, 143, 5855. 33835780 10.1021/jacs.1c00852

[122] M. Cha , P. Da , J. Wang , W. Wang , Z. Chen , F. Xiu , G. Zheng , Z. S. Wang , J. Am. Chem. Soc. 2016, 138, 8581. 27345104 10.1021/jacs.6b04519

[123] M. Que , Z. Dai , H. Yang , H. Zhu , Y. Zong , W. Que , N. P. Padture , Y. Zhou , O. Chen , ACS Energy Lett. 2019, 4, 1970.

[124] L. Xie , P. Vashishtha , T. M. Koh , P. C. Harikesh , N. F. Jamaludin , A. Bruno , T. J. N. Hooper , J. Li , Y. F. Ng , S. G. Mhaisalkar , N. Mathews , Adv. Mater. 2020, 32, 2003296. 10.1002/adma.202003296 32856340

[125] H. Xu , J. Duan , Y. Zhao , Z. Jiao , B. He , Q. Tang , J. Power Sources 2018, 399, 76.

[126] Y. Zhao , J. Duan , H. Yuan , Y. Wang , X. Yang , B. He , Q. Tang , Sol. RRL 2019, 3, 1800284.

[127] C. Jiang , J. Yao , P. Huang , R. Tang , X. Wang , X. Lei , H. Zeng , S. Chang , H. Zhong , H. Yao , C. Zhu , T. Chen , Cell Rep. Phys. Sci. 2020, 1, 100001.

[128] Z. Zhang , Z. Chen , J. Zhang , W. Chen , J. Yang , X. Wen , B. Wang , N. Kobamoto , L. Yuan , J. A. Stride , G. J. Conibeer , R. J. Patterson , S. Huang , Adv. Energy Mater. 2017, 7, 1601773.

[129] N. Guijarro , L. Yao , F. Le Formal , R. A. Wells , Y. Liu , B. P. Darwich , L. Navratilova , H. H. Cho , J. H. Yum , K. Sivula , Angew Chem., Int. Ed. 2019, 58, 12696. 10.1002/anie.201906803 31328858

[130] Y. Wang , B. Jia , J. Wang , P. Xue , Y. Xiao , T. Li , J. Wang , H. Lu , Z. Tang , X. Lu , F. Huang , X. Zhan , Adv. Mater. 2020, 32, 2002066. 10.1002/adma.202002066 32529680

[131] Y. Li , S. Zhou , Z. Xiong , M. Qin , K. Liu , G. Cai , H. Wang , S. Zhao , G. Li , Y.‐J. Hsu , J. Xu , X. Lu , ACS Appl. Energy Mater. 2020, 3, 11359.

[132] H. Zai , C. Zhu , H. Xie , Y. Zhao , C. Shi , Z. Chen , X. Ke , M. Sui , C. Chen , J. Hu , Q. Zhang , Y. Gao , H. Zhou , Y. Li , Q. Chen , ACS Energy Lett. 2017, 3, 30.

[133] Y. Gao , Y. Wu , H. Lu , C. Chen , Y. Liu , X. Bai , L. Yang , W. W. Yu , Q. Dai , Y. Zhang , Nano Energy 2019, 59, 517.

[134] X. X. Feng , X. D. Lv , Q. Liang , J. Cao , Y. Tang , ACS Appl. Mater. Interfaces 2020, 12, 16236. 32176484 10.1021/acsami.9b21348

[135] X. Zheng , J. Troughton , N. Gasparini , Y. Lin , M. Wei , Y. Hou , J. Liu , K. Song , Z. Chen , C. Yang , B. Turedi , A. Y. Alsalloum , J. Pan , J. Chen , A. A. Zhumekenov , T. D. Anthopoulos , Y. Han , D. Baran , O. F. Mohammed , E. H. Sargent , O. M. Bakr , Joule 2019, 3, 1963.

[136] W. Yang , R. Su , D. Luo , Q. Hu , F. Zhang , Z. Xu , Z. Wang , J. Tang , Z. Lv , X. Yang , Y. Tu , W. Zhang , H. Zhong , Q. Gong , T. P. Russell , R. Zhu , Nano Energy 2020, 67, 104189.

[137] D. Parobek , B. J. Roman , Y. Dong , H. Jin , E. Lee , M. Sheldon , D. H. Son , Nano Lett. 2016, 16, 7376. 27797528 10.1021/acs.nanolett.6b02772

[138] a) O. Malinkiewicz , C. Roldán‐Carmona , A. Soriano , E. Bandiello , L. Camacho , M. K. Nazeeruddin , H. J. Bolink , Adv. Energy Mater. 2014, 4, 1400345;

[139] A. O. El‐Ballouli , O. M. Bakr , O. F. Mohammed , Chem. Mater. 2019, 31, 6387.

[140] a) O. Y. Gong , Y. Kim , D. H. Kim , G. S. Han , S. Jeong , H. S. Jung , ACS Energy Lett. 2020, 5, 1032;

[141] D. Moghe , L. Wang , C. J. Traverse , A. Redoute , M. Sponseller , P. R. Brown , V. Bulović , R. R. Lunt , Nano Energy 2016, 28, 469.

[142] a) C. Liu , L. Huang , X. Zhou , X. Wang , J. Yao , Z. Liu , S. F. Liu , W. Ma , B. Xu , Sci. Bull. 2021, 66, 1419; 10.1016/j.scib.2021.03.018 36654368

[143] J. Tang , K. W. Kemp , S. Hoogland , K. S. Jeong , H. Liu , L. Levina , M. Furukawa , X. Wang , R. Debnath , D. Cha , Nat. Mater. 2011, 10, 765. 21927006 10.1038/nmat3118

[144] A. Banerjee , F. S. Liu , D. Beglau , T. Su , G. Pietka , J. Yang , S. Guha , IEEE J. Photovoltaics 2012, 2, 104.

[145] V. Pecunia , L. G. Occhipinti , A. Chakraborty , Y. Pan , Y. Peng , APL Mater. 2020, 8, 100901.

[146] J. Jean , J. Xiao , R. Nick , N. Moody , M. Nasilowski , M. Bawendi , V. Bulović , Energy Environ. Sci. 2018, 11, 2295.

[147] V. Benda , L. Cerna , Heliyon 2020, 6, 05666. 10.1016/j.heliyon.2020.e05666 PMC7750313 33364478

